# Sexualized drug use among gay men and other men who have sex with men in Latin America: A description of the phenomenon based on the results of LAMIS-2018

**DOI:** 10.1371/journal.pone.0287683

**Published:** 2023-10-19

**Authors:** Cristian Lisboa, Valeria Stuardo, Cinta Folch

**Affiliations:** 1 Escuela de Salud Pública, Universidad de Chile, Santiago, Chile; 2 Instituto de Salud Pública, Universidad Andrés Bello, Santiago, Chile; 3 Centro de Estudios Epidemiológicos sobre las Infecciones de Transmisión Sexual y SIDA de Cataluña (CEEISCAT), Cataluña, España; 4 Centro de Investigación Biomédica en Red de Epidemiología y Salud Pública (CIBERESP), Madrid, España; University of Technology Sydney, AUSTRALIA

## Abstract

**Introduction:**

Sexualized drug use (SDU) to enhance and extend sexual relations may involve risks of substances abuse (intoxication, interactions and overdose) and higher exposure to HIV and other sexually transmitted infections. There are inconsistencies in the methodology and findings of previous research on SDU in Latin America (LA), and more studies are required. The purpose of this research was to characterize SDU in gay men and other men who have sex with men from 18 LA countries, and describe the aspects by comparing people who practice and do not practice SDU, at the general and country levels.

**Material and methods:**

Cross-sectional study based on the data collected by LAMIS-2018. Dependent variable was SDU (last 12 months), and the independent variables were: drug use (in any context/in sexual context), sociodemographic, socioepidemiological, and psychosocial aspects. A descriptive analysis was carried out, comparing those who practiced and did not practice SDU.

**Results:**

LAMIS-2018 included 64,655 participants, averaging 30 years of age. 13.6% declared having practiced SDU (6.6% with multiple partners). In the last sexual encounter the most commonly used drugs were cannabis (9.3%), poppers (6%), and Viagra (5.4%), and in the last encounter with multiple partners, poppers (19.7%), cannabis (17%), and Viagra (13.2%). HIV diagnosis was reported by 27% of people practicing SDU, vs. 14.3% in the other group. Severe anxiety-depression symptoms were more common among people practicing SDU (9.2% vs. 7%), as were the episodes of homophobic intimidation (52.6% vs. 48.2%), insults (34.4% vs. 28.6%), and aggression (4.1% vs. 3.0%).

**Conclusions:**

SDU was reported by a high percentage of people, with a predominance of the use of drugs related to sexual practice, and others for recreational use. Aspects described as the higher proportion of self-reported HIV diagnosis and severe symptoms of anxiety-depression among those who practiced SDU, show that is necessary to implement preventive strategies to reduce the harmful impacts that can sometimes result from this practice, including harm reduction policies, promote access to mental health services and support in situations of homophobia and stigma.

## Introduction

### Drug use in the Latin American context

Drug use is a multidimensional phenomenon that covers a wide spectrum of scenarios, ranging from recreational use of psychoactive substances (PS) to their abuse [1]. Drug consumption in Latin America (LA) is mainly seen in young men, with cannabis and cocaine being the most prevalent illicitly used drugs [2]. In the case of recreational cannabis use, consumption in men and women has increased during the last decades (although the gender gap persists), which could be related to a decrease in the perception of risk or an increase in the perception of ease access [3]. Historically, the approach toward drug use has been correlated with socio-political criteria [4] with a wide range of perspectives, such as following the philosophy of prevention [5], penalizing consumption using a prohibitionist legal approach [6]; undergoing harm reduction [7] focusing on interventions aimed at reducing health damage [8], via syringe exchange programs [9], supervising consumption rooms [10, 11] and providing methadone treatments [12], among others methods. Perspectives such as management of risk and pleasure that emphasizes the pleasure associated with drug use have recently been incorporated [13] with the developed strategies to minimize the risks associated to drug consumption [14], regulate consumption behavior using knowledge, self-control, and self-efficacy [15]. The “fighting against drugs” policy has prevailed in LA [16], but it has not achieved the expected results [17]. However, there has recently been a certain degree of open-mindedness toward the decriminalization of the possession and consumption of certain substances (mainly for cannabis) [18]. Uruguay stands out for regulating and legalizing cannabis [19], while the few harm reduction strategies have had to coexist with the prevailing prohibitionism [20]; strategies that focus primarily on smokable cocaine (crack and cocaine base paste) [21].

### Sexualized drug use (chemsex)

Gay men and other men who have sex with men (MSM) constitute a key population in efforts against HIV, where the transmission of the virus is favored by aspects of sexual health, individual behaviors such as alcohol and drug consumption and phenomena such as discrimination and criminalization [22]. Chemsex is defined as the intentional use of drugs by gay men and other MSM to enhance and extend their sexual relations (often with multiple partners) [23], entailing the risks of substances abuse (intoxication, interactions, and overdose) and higher exposure to HIV and other sexually transmitted infections (STIs) [24]. Furthermore, studies have indicated that people practicing sexualized drug use (SDU) are more likely to have sex with multiple partners and more often practices such as “fisting,” toy exchange, and “slamsex,” which is defined as intravenous drug injection before or during sexual activity [25].

Determining the prevalence of this phenomenon in LA is a complex task, owing to the lack of information and the methodological differences among studies that hinder the comparison of their results. In a study conducted in Colombia on 766 people (78% men and 21% women), among whom 80% people were homo/bisexual, 54% participants reported engaging in “chemsex” for more than a year; poppers, MDMA, cocaine, and ketamine were the most commonly used substances [26]. In Brazil, a study on the vulnerability to HIV of MSM who were over 50 years of age and used dating apps estimated that 11.7% had practiced SDU in the last 30 days [27]. Further, a study assessing SDU in Brazil during the coronavirus disease pandemic found that among the surveyed 1,651 MSM, 84.5% practiced SDU in their sexual relationships during the periods of confinement, especially with casual partners (95%) [28]. In Argentina, a study conducted on the general population (n = 2,924) including 14.9% MSM, revealed that 3.9% participants practiced chemsex, data that was approximately four times higher than the overall prevalence reported (1.1%) [29]. Among 4,945 gay men an other MSM who were surveyed in Chile, 24% reported practicing SDU and 10.5% declared practicing SDU with multiple partners in the last 12 months; cannabis, popper, Viagra, and cocaine were the most commonly used drugs [30].

This research used the term “sexualized drug use” as it allows a broader exploration of the phenomenon, regardless of the type of drugs consumed. From the perspective of the social determinants of health [31], multiple factors should be considered while studying the SDU in LA context, due to the deployment of axes of inequality that determine differences among people in a variety of aspects such as education, employment, living conditions, work, etc. The differences create a social gradient that entails health consequences for those who practice SDU [32], for which the analysis of these sociodemographic aspects is necessary for the study of a phenomenon that is multidimensional. In addition, it is important to consider the influence of persistent social phenomena in the region, such as the stigma associated with HIV, gender identity and sexual orientation, which may be related with the use of PS and a greater burden of disease in gay men and other MSM. These factors create a syndemic process from the interacting phenomena that becomes mutually enhancing, affecting the health of the people [33].

The purpose of this research was to characterize SDU in gay men and other MSM from 18 LA countries, and describe the sociodemographic, socioepidemiological and psychosocial aspects by comparing people who practice and do not practice SDU, at the general and country levels. Consequently, this research hopes to contribute to public health providing detailed information on an emerging phenomenon whose characteristics and impact on the population are still unknown, constituting a possible input for future health policies in the region.

## Material and methods

### Study design

This was a cross-sectional study based on the data collected by the Latin America Men who have Sex with Men Internet Survey (LAMIS) 2018 [34], the first online survey on psycho-socio-sexual health of gay men and other MSM. The survey was conducted in 18 LA countries: Argentina, Bolivia, Brazil, Chile, Colombia, Costa Rica, Ecuador, El Salvador, Guatemala, Honduras, Mexico, Nicaragua, Panama, Paraguay, Peru, Suriname, Uruguay and Venezuela. The survey was promoted by the Red Iberoamericana de Estudios en Hombres Gay, otros Hombres que tienen Sexo con Hombres y Personas Trans (RIGHT Plus), the School of Psychology and Neuroscience, Maastricht University (Netherlands), the Department for Infectious Disease Epidemiology, Robert Koch Institute (Germany), and Sigma Research at the London School of Hygiene and Tropical Medicine (LSHTM) (UK).

### Population and inclusion criteria

The included population comprised gay men and other MSM who were 18 or older and resided in one of the 18 participating countries.

### Recruitment and methodology for information collection

The online questionnaire used for the study was adapted from the EMIS-2017 [35]. The questionnaire comprised closed and multiple choice questions that were aimed at obtaining sociodemographic information and indicators for monitoring and planning prevention programs for health and risk behavior of gay men and other MSM. The survey was available in three different languages: Spanish, Portuguese, and Dutch.

Promotion and recruitment of participants was carried out from January 24 to May 13, 2018, using mobile dating applications for men, web pages, social networks, clinics, NGOs, organizations and leisure places commonly visited by the study population. For online promotion, different images and banners (dynamic and static) were created in the three study languages using a common base graphic that was modified according to the language and colors used in each participating country. In addition, offline promotion was carried out using printed materials (posters and cards) that were displayed in the gay leisure venues (discos, saunas, etc.), depending on the country. People reached by recruitment strategies gained access to a link that directed them to the survey web page. The first page contained the informed consent providing study information, including a brief summary of the study purpose and its relevance, institutions involved, inclusion and exclusion criteria, benefits and potential harms, estimated time to complete the survey, and data management. Before moving on to the survey questions, all participants must have stated that they understood the nature and purpose of the study and that they consented to take part in it. After consent, the only compulsory questions were the first ones about age, sexual identity, sex assigned at birth and country of residence, to verify inclusion and exclusion criteria.

### Dependent variable

SDU practice was defined based on the question “When was the last time you used drugs to make your sexual relations more intense or last longer?,” with eight options as answers: last 24 hours, last 7 days, last 4 weeks, last 6 months, last 12 months, last 5 years, more than 5 years ago, and never. For the analysis, all categories were merged to obtain a qualitative/dichotomous variable, where 0 indicated “Never or more than 12 months ago” and 1 indicated “In the last 12 months”.

### Independent variables

- *Drugs use in any context (recreational use)*: alcohol consumption (last 12 months), alcohol abuse (assessed using CAGE4 questionnaire, which is a screening tool widely used to measure alcohol abuse and dependence in a population [36]), drugs consumed in any context (last 12 months), and injection drug use (sometime in life).
- *Drugs use in the sexual context*: SDU with multiple partners at the same time (last 12 months), place where the SDU with multiple partners occurred, and drug use during the last sexual encounter (either with a casual partner or as part of a threesome involving the steady partner or multiple partners).
- *Sociodemographic aspects*: age, migratory status, highest educational qualification, occupation, and self-perception of economic income.
- *Socioepidemiological aspects*: stable partner, casual partners (last 12 months), number of casual sexual partners (last 12 months), number of casual sexual encounters without using a condom (last 12 months), satisfaction with sexual life, payments received (money, gifts, or favors) in exchange for sex with men (last 12 months), self-reported HIV diagnosis, use of pre-exposure prophylaxis (PrEP) (sometime in life), and positive diagnosis for hepatitis C, syphilis, gonorrhea, chlamydia, and anal or genital warts (condyloma), sometime in life.
- *Psychosocial aspects*: anxiety-depression symptoms (assessed using “Patient Health Questionnaire”—PHQ-4 [37], social support (assessed using two subscales of social provision regarding “Social integration” and “Reliable alliance” designed by Cutrona and Russell [38] to detect the lack of social connection [39], internalized homonegativity (assessed using “Reactions to Homosexuality Scale” [40], homophobic intimidation (last 12 months), homophobic insults (last 12 months), and homophobic aggressions (last 12 months).

### Data analysis

A descriptive analysis was performed to estimate the general and country-level prevalence of SDU in the last 12 months. Subsequently, the frequency and distribution of the independent variables in each study group (SDU Yes/No) were determined. The categorical, continuous, and discrete variables were compared using Chi-square, Student’s T, and Mann–Whitney U tests, respectively. Outcomes were recorded in tables for all samples and participating countries, distributing in columns the respective values for those who practiced SDU in the last 12 months, those who did not practice SDU in the las 12 months and, the p-value obtained from the comparison between both groups.

### Ethical considerations

The LAMIS-2018 study obtained the ethical approvals for its execution at the regional level by the committees of the Universidad Peruana Cayetano Heredia (612-19-17), Escuela de Salud Pública Salvador Allende, School of Medicine, Universidad de Chile (009–2017), Santa Casa de Misericórdia de São, Brazil (2,457,744), Comité Nacional de Ética en Salud, Guatemala (National Committee for Health Ethics) (39–2017), and the Faculty of Psychology and Neuroscience of the University of Maastricht, Netherlands (186-01-12-2017).

## Results

LAMIS-2018 included 64,655 participants, wherein most participants were from Brazil (n = 18,139), Mexico (n = 14,957), Colombia (n = 8,208), Argentina (n = 5,504), and Chile (n = 4,945). The estimated recruitment rate revealed that there were 3.3 participants for every 10,000 men included in the study and were aged between 15 and 65 years (range: 1.9–11.7, depending on the country).

### Drug use in any context (recreational use)

The prevalence of alcohol consumption in the last 12 months overall was 90.1% (Tables 1 and 2), which was similar to the prevalence at country level, with the exception of El Salvador (78.2%). The overall prevalence of alcohol dependence was 21.1%; Bolivia (36%) and Nicaragua (35.3%) had the highest prevalence rates among the 18 countries. The overall prevalence of drug use (sometime in life) was 40.3%, with Chile (56.3%) and Uruguay (52.6%) being the predominant countries. The prevalence of injectable drug use was 0.9%, and the country-level prevalence ranged within 0% - 1.4%. In terms of drugs consumption in any context (recreational use), the most frequently used drug was cannabis (29.7%) and Chile and Uruguay were its predominant users (49.9% and 44.7%, respectively). The use of cannabis was followed by nitrites (poppers) (17.7%) and was predominantly used in Colombia (31.5%). Globally, cocaine was used by 9.5% participants; approximately 12% participants used cocaine in Uruguay, Brazil, and Chile. Among synthetic drugs, consumption of ecstasy as pills and crystals (11.9%), synthetic cannabinoids (6.2%), and LSD (5.8%) were predominantly used overall. At country level, high consumption of synthetic cannabinoids in Chile (15.5%), ecstasy pills in Brazil (12.8%), and LSD in Uruguay (12.1%) were observed.

**Table 1:** Descriptive analysis of drug use in any context (recreational use) and sexualized drug use, in total sample and each country participating in LAMIS-2018 (part one).

VARIABLE	TOTAL SAMPLE	Argentina	Bolivia	Brazil	Chile	Colombia	Costa Rica	Ecuador	El Salvador	Guatemala
	(N = 64655)	(N = 5504)	(N = 748)	(N = 18139)	(N = 4945)	(N = 8208)	(N = 1012)	(N = 1440)	(N = 572)	(N = 1157)
	n (%)	n (%)	n (%)	n (%)	n (%)	n (%)	n (%)	n (%)	n (%)	n (%)
**Consumption of alcohol** (last 12 months)	57967 (90.1)	4977 (90.8)	659 (88.7)	16248 (90.0)	4576 (92.7)	7465 (91.4)	888 (88.0)	1294 (90.2)	444 (78.2)	954 (82.8)
**Alcohol dependence** (CAGE4)	13539 (21.1)	734 (13.5)	266 (36.0)	4454 (24.9)	1167 (23.7)	1530 (18.9)	170 (16.9)	380 (26.7)	118 (20.8)	287 (25.2)
**Prevalence of drug use** (at least once in lifetime)	25786 (40.3)	2217 (40.6)	232 (31.3)	8885 (49.4)	2769 (56.3)	2684 (33.1)	446 (44.6)	476 (33.4)	133 (23.5)	318 (27.9)
**Drug use in any context** (last 12 months)										
Cannabis	18989 (29.7)	1810 (33.2)	166 (22.4)	6508 (36.2)	2453 (49.9)	1931 (23.8)	356 (35.6)	358 (25.1)	77 (13.6)	209 (18.4)
Poppers (nitrite inhalants)	11356 (17.7)	684 (12.5)	50 (6.7)	1539 (8.5)	1446 (29.3)	2571 (31.5)	203 (20.1)	207 (14.5)	55 (9.7)	198 (17.3)
Viagra (cialis)	9410 (14.7)	813 (14.9)	94 (12.7)	2793 (15.5)	854 (17.3)	1243 (15.2)	95 (9.4)	127 (8.9)	50 (8.8)	129 (11.2)
Synthetic cannabinoids	3932 (6.2)	293 (5.4)	49 (6.6)	427 (2.4)	763 (15.5)	982 (12.1)	106 (10.6)	127 (8.9)	17 (3.0)	43 (3.8)
Sedatives (valium)	8247 (12.8)	1001 (18.3)	83 (11.2)	3551 (19.7)	721 (14.6)	394 (4.8)	84 (8.4)	90 (6.3)	35 (6.2)	83 (7.2)
Cocaine	6086 (9.5)	413 (7.6)	38 (5.1)	2211 (12.3)	571 (11.6)	850 (10.5)	91 (9.1)	98 (6.9)	48 (8.5)	109 (9.6)
LSD	3754 (5.8)	424 (7.8)	32 (4.3)	1610 (9.0)	377 (7.7)	469 (5.8)	67 (6.7)	47 (3.3)	3 (0.5)	28 (2.5)
Ecstasy in the form of a pill	4696 (7.4)	420 (7.7)	18 (2.4)	2297 (12.8)	337 (6.9)	544 (6.7)	57 (5.7)	39 (2.7)	6 (1.1)	29 (2.5)
Ecstasy in the form of a crystal (MDMA)	2903 (4.5)	218 (4.0)	10 (1.3)	1370 (7.6)	224 (4.6)	288 (3.6)	103 (10.3)	25 (1.8)	5 (0.9)	24 (2.1)
Ketamine	1852 (2.9)	88 (1.6)	7 (0.9)	1185 (6.6)	126 (2.6)	235 (2.9)	19 (1.9)	6 (0.4)	2 (0.3)	5 (0.4)
GHB/GBL	1144 (1.8)	81 (1.5)	3 (0.4)	626 (3.5)	115 (2.3)	71 (0.9)	9 (0.9)	9 (0.6)	0 (0.0)	9 (0.8)
Amphetamines	1346 (2.1)	447 (8.2)	3 (0.4)	399 (2.2)	79 (1.6)	125 (1.5)	8 (0.8)	8 (0.6)	2 (0.3)	10 (0.9)
Crack cocaine	557 (0.9)	22 (0.4)	5 (0.7)	82 (0.5)	41 (0.8)	69 (0.8)	10 (1.0)	13 (0.9)	8 (1.4)	15 (1.3)
Crystal Methamphetamines	1005 (1.6)	67 (1.2)	4 (0.5)	240 (1.3)	35 (0.7)	79 (1.0)	8 (0.8)	7 (0.5)	1 (0.2)	12 (1.0)
Heroine	313 (0.5)	13 (0.2)	1 (0.1)	121 (0.7)	20 (0.4)	38 (0.5)	3 (0.3)	7 (0.5)	3 (0.5)	5 (0.4)
Mephedrone	172 (0.3)	4 (0.1)	2 (0.3)	69 (0.4)	17 (0.4)	28 (0.4)	0 (0.0)	2 (0.1)	0 (0.0)	2 (0.2)
Synthetic stimulants other than mephedrone (tusi)	414 (0.7)	25 (0.5)	5 (0.7)	101 (0.6)	37 (0.7)	124 (1.5)	8 (0.8)	4 (0.3)	0 (0.0)	7 (0.6)
**Injected drug use** (at least once in lifetime)	565 (0.9)	25 (0.5)	4 (0.5)	257 (1.4)	32 (0.6)	44 (0.5)	6 (0.6)	8 (0.6)	2 (0.4)	4 (0.4)
**Sexualized drug use (SDU)** (last 12 months)	8690 (13.6)	703 (12.9)	75 (10.1)	2321 (12.9)	1190 (24.2)	1304 (16.1)	177 (17.7)	142 (10.0)	26 (4.6)	103 (9.0)
**SDU with multiple partners** (last 12 months)	4220 (6.6)	340 (6.2)	27 (3.7)	1100 (6.1)	515 (10.5)	661 (8.2)	88 (8.8)	62 (4.4)	15 (2.7)	55 (4.8)
**Location of group SDU**	(N = 4218)	(N = 340)	(N = 27)	(N = 1099)	(N = 515)	(N = 661)	(N = 88)	(N = 62)	(N = 15)	(N = 55)
(Where did the sexual encounter take place?)										
My home	1090 (25.8)	106 (31.2)	9 (33.3)	290 (26.4)	165 (32.0)	143 (21.6)	28 (31.8)	17 (27.4)	3 (20.0)	11 (20.0)
Home of another partners	1674 (39.7)	151 (44.4)	11 (40.7)	401 (36.5)	243 (47.2)	274 (41.4)	37 (42.1)	29 (46.8)	8 (53.3)	21 (38.2)
Hotel room	480 (11.4)	18 (5.3)	5 (18.5)	119 (10.8)	27 (5.2)	58 (8.8)	7 (7.9)	10 (16.1)	3 (20.0)	12 (21.8)
Club or back room	337 (7.9)	29 (8.5)	0 (0.0)	79 (7.2)	16 (3.1)	68 (10.3)	4 (4.5)	0 (0.0)	0 (0.0)	5 (9.1)
Others	637 (15.1)	36 (10.6)	2 (7.4)	210 (19.1)	64 (12.4)	118 (17.8)	12 (13.6)	6 (9.7)	1 (6.6)	6 (10.9)

**Table 2:** Descriptive analysis of drug use in any context (recreational use) and sexualized drug use, in total sample and each country participating in LAMIS-2018 (part two).

VARIABLE	Honduras	México	Nicaragua	Panamá	Paraguay	Perú	Suriname	Uruguay	Venezuela
	(N = 646)	(N = 14957)	(N = 534)	(N = 759)	(N = 591)	(N = 2025)	(N = 216)	(N = 771)	(N = 2431)
	n (%)	n (%)	n (%)	n (%)	n (%)	n (%)	n (%)	n (%)	n (%)
**Consumption of alcohol** (last12 months)	536 (83.2)	13391 (90.0)	458 (86.3)	653 (86.4)	509 (86.7)	1825 (91.0)	190 (89.2)	696 (90.6)	2204 (91.0)
**Alcohol dependence** (CAGE 4)	173 (27.1)	2965 (20.1)	187 (35.3)	143 (19.1)	88 (15.2)	471 (23.8)	32 (15.2)	89 (11.7)	285 (11.9)
**Prevalence of drug use** (at least once in lifetime)	196 (30.7)	5371 (36.3)	128 (24.1)	198 (26.4)	172 (29.6)	523 (26.2)	81 (38.4)	402 (52.6)	555 (23.2)
**Drug use in any context** (last 12 months)									
Cannabis	127 (19.9)	3583 (24.2)	89 (16.8)	129 (17.2)	128 (22.1)	358 (17.9)	63 (29.9)	341 (44.7)	303 (12.7)
Poppers (nitrite inhalants)	79 (12.3)	3574 (24.0)	33 (6.2)	146 (19.3)	74 (12.6)	223 (11.1)	58 (27.2)	100 (13.1)	116 (4.8)
Viagra (cialis)	44 (6.8)	2296 (15.4)	33 (6.2)	77 (10.2)	68 (11.6)	249 (12.4)	30 (14.1)	91 (11.9)	324 (13.4)
Synthetic cannabinoids	38 (6.0)	697 (4.7)	20 (3.7)	50 (6.7)	22 (3.8)	68 (3.4)	6 (2.9)	44 (5.7)	180 (7.5)
Sedatives (valium)	50 (7.8)	1280 (8.6)	76 (14.3)	61 (8.1)	79 (13.5)	236 (11.8)	33 (15.5)	177 (23.2)	213 (8.8)
Cocaine	65 (10.2)	1188 (8.0)	37 (7.0)	44 (5.9)	59 (10.1)	94 (4.7)	12 (5.7)	94 (12.4)	64 (2.7)
LSD	12 (1.9)	517 (3.5)	7 (1.3)	14 (1.9)	16 (2.8)	21 (1.0)	0 (0.0)	92 (12.1)	18 (0.7)
Ecstasy in the form of a pill	9 (1.4)	750 (5.1)	8 (1.5)	16 (2.1)	19 (3.3)	25 (1.2)	19 (9.0)	76 (10.0)	27 (1.1)
Ecstasy in the form of a crystal (MDMA)	5 (0.8)	489 (3.3)	5 (0.9)	13 (1.7)	12 (2.1)	24 (1.2)	14 (6.6)	57 (7.5)	17 (0.7)
Ketamine	3 (0.5)	118 (0.8)	3 (0.6)	3 (0.4)	3 (0.5)	9 (0.4)	4 (1.9)	23 (3.0)	13 (0.5)
GHB/GBL	0 (0.0)	182 (1.2)	2 (0.4)	7 (0.9)	2 (0.3)	9 (0.4)	5 (2.4)	5 (0.7)	9 (0.4)
Amphetamines	5 (0.8)	178 (1.2)	2 (0.4)	5 (0.7)	9 (1.5)	10 (0.5)	1 (0.5)	50 (6.5)	5 (0.2)
Crack cocaine	9 (1.4)	238 (1.6)	8 (1.5)	4 (0.5)	4 (0.7)	17 (0.8)	5 (2.4)	3 (0.4)	4 (0.2)
Crystal Methamphetamines	2 (0.3)	490 (3.3)	5 (0.9)	5 (0.7)	2 (0.3)	10 (0.5)	8 (3.8)	26 (3.4)	4 (0.2)
Heroine	3 (0.5)	84 (0.6)	1 (0.2)	3 (0.4)	0 (0.0)	2 (0.1)	1 (0.5)	4 (0.5)	4 (0.2)
Mephedrone	0 (0.0)	36 (0.2)	2 (0.4)	1 (0.1)	0 (0.0)	4 (0.2)	1 (0.5)	2 (0.3)	2 (0.1)
Synthetic stimulants other than mephedrone (tusi)	0 (0.0)	76 (0.5)	1 (0.2)	2 (0.3)	2 (0.3)	6 (0.3)	2 (0.9)	8 (1.0)	6 (0.2)
**Injected drug use** (at least once in lifetime)	2 (0.3)	132 (0.9)	0 (0.0)	4 (0.5)	6 (1.0)	9 (0.5)	2 (1.0)	9 (1.2)	19 (0.8)
**Sexualized drug use (SDU)** (last 12 months)	52 (8.2)	2009 (13.6)	31 (5.9)	57 (7.6)	65 (11.2)	158 (7.9)	23 (10.9)	135 (17.7)	119 (5.0)
**SDU with multiple partners** (last 12 months)	22 (3.5)	1078 (7.3)	10 (1.9)	23 (3.1)	37 (6.4)	66 (3.3)	12 (5.7)	59 (7.7)	50 (2.1)
**Location of group SDU**	(N = 22)	(N = 1077)	(N = 10)	(N = 23)	(N = 37)	(N = 66)	(N = 12)	(N = 59)	(N = 50)
(Where did the sexual encounter take place?)	** **								
My home	8 (36.4)	243 (22.6)	3 (30.0)	1 (4.3)	6 (16.2)	17 (25.8)	7 (58.3)	17 (28.8)	16 (32.0)
Home of another partners	8 (36.4)	390 (36.2)	4 (40.0)	9 (39.1)	16 (42.2)	22 (33.3)	0 (0.0)	28 (47.4)	22 (44.0)
Hotel room	3 (13.6)	176 (16.3)	3 (30.0)	7 (30.4)	6 (16.2)	14 (21.2)	3 (25.0)	3 (5.1)	6 (12.0)
Club or back room	0 (0.0)	124 (11.5)	0 (0.0)	2 (8.7)	2 (5.4)	4 (6.1)	1 (8.3)	2 (3.4)	1 (2.0)
Others	3 (13.6)	144 (13.3)	0 (0.0)	4 (17.4)	7 (18.9)	9 (13.6)	1 (8.3)	9 (15.2)	5 (10.0)

### SDU

**Fig 1:**
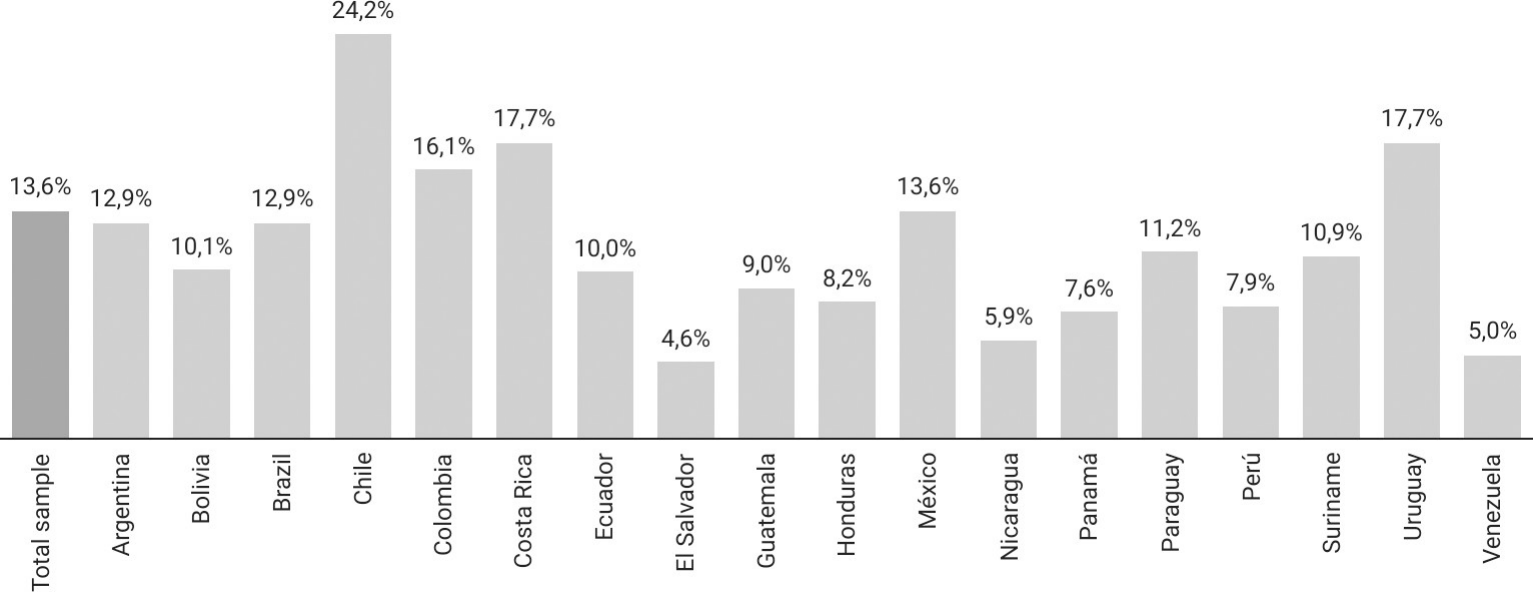
Prevalence of SDU (last 12 months) in total sample and each country participating in LAMIS-2018.

**Fig 2:**
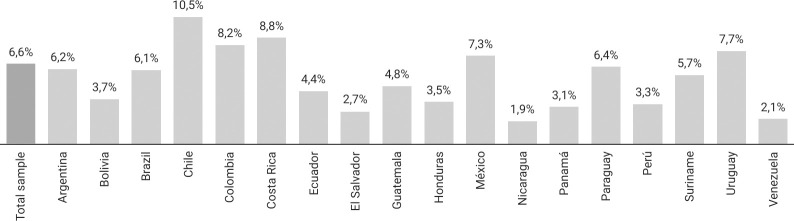
Prevalence of SDU with multiple partners (last 12 months) in total sample and each country participating in LAMIS-2018.

Of the total number of study participants (N = 64,655), 13.6% declared having practiced SDU in the last 12 months (Tables 1 and 2). Variation in SDU practices was observed while disaggregating the data at country level; Chile (24.2%), Costa Rica (17.7%), and Uruguay (17.7%) exhibited the highest prevalence of SDU (Fig 1). The practice of SDU with multiple partners had a overall prevalence of 6.6%; Chile (10.5%), Costa Rica (8.8%), and Colombia (8.2%) had the highest country-level prevalences (Fig 2). Private homes were the commonly used places for group encounters, wherein the group used the home of one of those involved in the group encounter (39.7%) or the respondent himself (25.8%).

Regarding drug use during the last casual sexual encounter with a partner or while being part of a threesome that involved a stable partner (Tables 3 and 4), the most commonly used drugs overall were cannabis (9.3%), poppers (6%), and Viagra (5.4%). At the country level, Chile and Suriname had the highest consumption rates for cannabis (21.8%) and poppers (13.2%), respectively. Viagra was most commonly used in Chile and Uruguay (7.2% and 7.1%, respectively). Regarding drug use in the last sexual encounter with multiple partners, poppers (19.7%), cannabis (17%), and Viagra (13.2%) were the most commonly used drugs. The use of cocaine and ecstasy (pill and crystal) was reported by 6.8% and 4.5% participants, respectively. At the country level, poppers was used by more than 30% participants from Chile, Colombia, and Mexico. Chile also stood out in the consumption of cannabis (33.7%) and cocaine (10.6%).

**Table 3:** Descriptive analysis of drug use before or during most recent sexual encounter among men surveyed, in total sample and each country participating in LAMIS-2018 (part one).

VARIABLE	TOTAL SAMPLE	Argentina	Bolivia	Brazil	Chile	Colombia	Costa Rica	Ecuador	El Salvador	Guatemala
	n (%)	n (%)	n (%)	n (%)	n (%)	n (%)	n (%)	n (%)	n (%)	n (%)
	(N = 39266)	(N = 3453)	(N = 450)	(N = 11145)	(N = 2949)	(N = 5141)	(N = 582)	(N = 873)	(N = 332)	(N = 709)
**With a casual partner or a threesome with a stable partner**										
Cannabis	3642 (9.3)	356 (10.3)	27 (6.0)	1038 (9.3)	644 (21.8)	486 (9.5)	92 (15.8)	58 (6.6)	12 (3.6)	29 (4.1)
Poppers (nitrite inhalants)	2351 (6.0)	92 (2.7)	10 (2.2)	190 (1.7)	269 (9.1)	608 (11.8)	48 (8.3)	44 (5.0)	7 (2.1)	56 (7.9)
Viagra (cialis)	2128 (5.4)	195 (5.7)	28 (6.2)	582 (5.2)	213 (7.2)	259 (5.0)	23 (4.0)	23 (2.6)	12 (3.6)	29 (4.1)
Synthetic cannabinoids	217 (0.6)	8 (0.2)	4 (0.9)	21 (0.2)	42 (1.4)	76 (1.5)	3 (0.5)	4 (0.5)	1 (0.3)	3 (0.4)
Sedatives (valium)	213 (0.5)	21 (0.6)	2 (0.4)	71 (0.6)	21 (0.7)	20 (0.4)	3 (0.5)	6 (0.7)	0 (0.0)	2 (0.3)
Cocaine	649 (1.7)	35 (1.0)	3 (0.7)	253 (2.3)	69 (2.3)	99 (1.9)	15 (2.6)	7 (0.8)	6 (1.8)	14 (2.0)
LSD	115 (0.3)	10 (0.3)	0 (0.0)	45 (0.4)	4 (0.1)	20 (0.4)	2 (0.3)	4 (0.5)	1 (0.3)	2 (0.3)
Ecstasy in the form of a pill	269 (0.7)	20 (0.6)	1 (0.2)	116 (1.0)	28 (1.0)	28 (0.5)	3 (0.5)	3 (0.3)	1 (0.3)	2 (0.3)
Ecstasy in the form of a crystal (MDMA)	105 (0.3)	5 (0.1)	0 (0.0)	42 (0.4)	9 (0.3)	10 (0.2)	7 (1.2)	1 (0.1)	0 (0.0)	1 (0.1)
Ketamine	135 (0.3)	4 (0.1)	0 (0.0)	75 (0.7)	10 (0.3)	20 (0.4)	6 (1.0)	1 (0.1)	1 (0.3)	0 (0.0)
GHB/GBL	83 (0.2)	8 (0.2)	0 (0.0)	52 (0.5)	8 (0.3)	5 (0.1)	0 (0.0)	0 (0.0)	0 (0.0)	1 (0.1)
Amphetamines	30 (0.1)	3 (0.1)	0 (0.0)	8 (0.1)	4 (0.1)	3 (0.1)	0 (0.0)	0 (0.0)	0 (0.0)	0 (0.0)
Crack cocaine	53 (0.1)	3 (0.1)	0 (0.0)	7 (0.1)	4 (0.1)	3 (0.1)	0 (0.0)	3 (0.3)	1 (0.3)	0 (0.0)
Crystal Methamphetamines	76 (0.2)	2 (0.1)	0 (0.0)	7 (0.1)	4 (0.1)	4 (0.1)	0 (0.0)	0 (0.0)	0 (0.0)	1 (0.1)
Heroine	11 (0.0)	0 (0.0)	0 (0.0)	4 (0.0)	2 (0.1)	2 (0.0)	0 (0.0)	0 (0.0)	0 (0.0)	0 (0.0)
Mephedrone	5 (0.0)	0 (0.0)	0 (0.0)	1 (0.0)	1 (0.0)	1 (0.0)	0 (0.0)	0 (0.0)	0 (0.0)	0 (0.0)
Synthetic stimulants other than mephedrone (tusi)	27 (0.1)	2 (0.1)	0 (0.0)	6 (0.1)	3 (0.1)	8 (0.2)	0 (0.0)	0 (0.0)	0 (0.0)	0 (0.0)
Have taken drugs but not sure which	72 (0.2)	7 (0.2)	2 (0.4)	22 (0.2)	7 (0.2)	7 (0.1)	2 (0.3)	1 (0.1)	0 (0.0)	1 (0.1)
Have not consumed any drugs	25820 (65.8)	2305 (66.8)	319 (70.9)	7474 (67.1)	1462 (49.6)	3470 (67.5)	359 (61.7)	622 (71.3)	254 (76.5)	488 (68.8)
**With multiple partners**	(N = 6799)	(N = 651)	(N = 57)	(N = 2270)	(N = 416)	(N = 939)	(N = 89)	(N = 114)	(N = 38)	(N = 95)
Cannabis	1155 (17.0)	122 (18.7)	6 (10.5)	329 (14.5)	140 (33.7)	181 (19.3)	21 (23.6)	20 (17.5)	5 (13.2)	12 (12.6)
Poppers (nitrite inhalants)	1339 (19.7)	72 (11.1)	1 (1.8)	169 (7.4)	146 (35.1)	316 (33.7)	28 (31.5)	20 (17.5)	8 (21.1)	22 (23.2)
Viagra (cialis)	894 (13.2)	81 (12.4)	4 (7.0)	276 (12.2)	87 (20.9)	114 (12.1)	10 (11.2)	6 (5.3)	6 (15.8)	15 (15.8)
Synthetic cannabinoids	110 (1.6)	7 (1.1)	0 (0.0)	32 (1.4)	17 (4.1)	37 (3.9)	1 (1.1)	0 (0.0)	0 (0.0)	1 (1.1)
Sedatives (valium)	75 (1.1)	7 (1.1)	0 (0.0)	26 (1.2)	8 (1.9)	5 (0.5)	0 (0.0)	2 (1.8)	0 (0.0)	0 (0.0)
Cocaine	465 (6.8)	35 (5.4)	1 (1.8)	175 (7.7)	44 (10.6)	59 (6.3)	7 (7.9)	6 (5.3)	4 (10.5)	10 (10.5)
LSD	88 (1.3)	8 (1.2)	1 (1.8)	23 (1.0)	6 (1.4)	16 (1.7)	1 (1.1)	2 (1.8)	0 (0.0)	1 (1.1)
Ecstasy in the form of a pill	201 (3.0)	13 (2.0)	1 (1.8)	89 (3.9)	8 (1.9)	22 (2.3)	3 (3.4)	3 (2.6)	1 (2.6)	4 (4.2)
Ecstasy in the form of a crystal (MDMA)	103 (1.5)	7 (1.1)	0 (0.0)	49 (2.2)	8 (1.9)	9 (1.0)	8 (9.0)	1 (0.9)	0 (0.0)	0 (0.0)
Ketamine	115 (1.7)	6 (0.9)	0 (0.0)	68 (3.0)	12 (2.9)	19 (2.0)	1 (1.1)	2 (1.8)	0 (0.0)	0 (0.0)
GHB/GBL	108 (1.6)	5 (0.8)	0 (0.0)	67 (3.0)	13 (3.1)	6 (0.6)	2 (2.3)	0 (0.0)	0 (0.0)	0 (0.0)
Amphetamines	37 (0.5)	6 (0.9)	0 (0.0)	13 (0.6)	3 (0.7)	5 (0.5)	2 (2.3)	0 (0.0)	0 (0.0)	0 (0.0)
Crack cocaine	47 (0.7)	2 (0.3)	0 (0.0)	8 (0.4)	0 (0.0)	4 (0.4)	2 (2.3)	0 (0.0)	0 (0.0)	0 (0.0)
Crystal Methamphetamines	88 (1.3)	3 (0.5)	0 (0.0)	16 (0.7)	1 (0.2)	4 (0.4)	2 (2.3)	0 (0.0)	0 (0.0)	0 (0.0)
Heroine	9 (0.1)	1 (0.2)	0 (0.0)	4 (0.2)	1 (0.2)	2 (0.2)	0 (0.0)	0 (0.0)	0 (0.0)	0 (0.0)
Mephedrone	6 (0.1)	1 (0.2)	0 (0.0)	3 (0.1)	0 (0.0)	1 (0.1)	0 (0.0)	0 (0.0)	0 (0.0)	1 (1.1)
Synthetic stimulants other than mephedrone (tusi)	28 (0.4)	1 (0.2)	0 (0.0)	7 (0.3)	2 (0.5)	10 (1.1)	0 (0.0)	0 (0.0)	0 (0.0)	0 (0.0)
Have taken drugs but not sure which	58 (0.9)	4 (0.6)	0 (0.0)	15 (0.7)	3 (0.7)	10 (1.1)	2 (2.3)	2 (1.8)	0 (0.0)	4 (4.2)
Have not consumed any drugs	3201 (47.0)	334 (51.3)	40 (70.2)	1196 (52.7)	111 (26.7)	442 (47.1)	39 (43.8)	58 (50.9)	22 (57.9)	48 (50.5)

**Table 4:** Descriptive analysis of drug use before or during most recent sexual encounter among men surveyed, in total sample and each country participating in LAMIS-2018 (part two).

VARIABLE	Honduras	México	Nicaragua	Panamá	Paraguay	Perú	Suriname	Uruguay	Venezuela
	n (%)	n (%)	n (%)	n (%)	n (%)	n (%)	n (%)	n (%)	n (%)
	(N = 358)	(N = 9136)	(N = 285)	(N = 431)	(N = 335)	(N = 1199)	(N = 91)	(N = 434)	(N = 1363)
**With a casual partner or a threesome with a stable partner**									
Cannabis	11 (3.1)	637 (7.0)	7 (2.5)	26 (6.0)	27 (8.1)	60 (5.0)	11 (12.1)	79 (18.2)	42 (3.1)
Poppers (nitrite inhalants)	15 (4.2)	899 (9.8)	6 (2.1)	25 (5.8)	12 (3.6)	28 (2.3)	12 (13.2)	14 (3.2)	16 (1.2)
Viagra (cialis)	10 (2.8)	556 (6.1)	5 (1.8)	19 (4.4)	18 (5.4)	49 (4.1)	4 (4.4)	31 (7.1)	72 (5.3)
Synthetic cannabinoids	0 (0.0)	28 (0.3)	0 (0.0)	5 (1.2)	1 (0.3)	5 (0.4)	0 (0.0)	3 (0.7)	13 (1.0)
Sedatives (valium)	3 (0.8)	37 (0.4)	3 (1.1)	3 (0.7)	5 (1.5)	5 (0.4)	0 (0.0)	7 (1.6)	4 (0.3)
Cocaine	4 (1.1)	107 (1.2)	2 (0.7)	4 (0.9)	8 (2.4)	10 (0.8)	0 (0.0)	7 (1.6)	6 (0.4)
LSD	0 (0.0)	21 (0.2)	0 (0.0)	1 (0.2)	1 (0.3)	1 (0.1)	0 (0.0)	3 (0.7)	0 (0.0)
Ecstasy in the form of a pill	0 (0.0)	60 (0.7)	1 (0.4)	0 (0.0)	0 (0.0)	2 (0.2)	1 (1.1)	2 (0.5)	1 (0.1)
Ecstasy in the form of a crystal (MDMA)	0 (0.0)	25 (0.3)	0 (0.0)	1 (0.2)	1 (0.3)	1 (0.1)	0 (0.0)	1 (0.2)	1 (0.1)
Ketamine	0 (0.0)	14 (0.2)	0 (0.0)	1 (0.2)	0 (0.0)	0 (0.0)	0 (0.0)	2 (0.5)	1 (0.1)
GHB/GBL	0 (0.0)	8 (0.1)	0 (0.0)	0 (0.0)	0 (0.0)	0 (0.0)	0 (0.0)	0 (0.0)	1 (0.1)
Amphetamines	0 (0.0)	9 (0.1)	0 (0.0)	1 (0.2)	1 (0.3)	0 (0.0)	0 (0.0)	0 (0.0)	1 (0.1)
Crack cocaine	0 (0.0)	27 (0.3)	0 (0.0)	1 (0.2)	0 (0.0)	1 (0.1)	0 (0.0)	1 (0.2)	2 (0.2)
Crystal Methamphetamines	0 (0.0)	52 (0.6)	0 (0.0)	1 (0.2)	0 (0.0)	0 (0.0)	0 (0.0)	2 (0.5)	3 (0.2)
Heroine	0 (0.0)	1 (0.0)	0 (0.0)	1 (0.2)	0 (0.0)	0 (0.0)	0 (0.0)	0 (0.0)	1 (0.1)
Mephedrone	0 (0.0)	1 (0.0)	1 (0.4)	0 (0.0)	0 (0.0)	0 (0.0)	0 (0.0)	0 (0.0)	0 (0.0)
Synthetic stimulants other than mephedrone (tusi)	0 (0.0)	5 (0.1)	0 (0.0)	1 (0.2)	1 (0.3)	0 (0.0)	0 (0.0)	0 (0.0)	1 (0.1)
Have taken drugs but not sure which	1 (0.3)	15 (0.2)	1 (0.4)	2 (0.5)	1 (0.3)	1 (0.1)	0 (0.0)	0 (0.0)	2 (0.2)
Have not consumed any drugs	260 (72.6)	5834 (63.9)	176 (61.8)	300 (69.6)	227 (67.8)	900 (75.1)	59 (64.8)	256 (59.0)	1055 (77.4)
**With multiple partners**	(N = 43)	(N = 1458)	(N = 34)	(N = 68)	(N = 67)	(N = 193)	(N = 16)	(N = 52)	(N = 199)
Cannabis	4 (9.3)	247 (16.9)	5 (14.7)	8 (11.8)	6 (9.0)	19 (9.8)	2 (12.5)	18 (34.6)	10 (5.0)
Poppers (nitrite inhalants)	6 (14.0)	480 (32.9)	2 (5.9)	17 (25.0)	8 (11.9)	22 (11.4)	8 (50.0)	7 (13.5)	7 (3.5)
Viagra (cialis)	4 (9.3)	234 (16.0)	0 (0.0)	4 (5.9)	5 (7.5)	20 (10.4)	4 (25.0)	9 (17.3)	15 (7.5)
Synthetic cannabinoids	1 (2.3)	9 (0.6)	0 (0.0)	0 (0.0)	1 (1.5)	0 (0.0)	0 (0.0)	0 (0.0)	4 (2.0)
Sedatives (valium)	1 (2.3)	23 (1.6)	0 (0.0)	1 (1.5)	0 (0.0)	1 (0.5)	0 (0.0)	1 (1.9)	0 (0.0)
Cocaine	2 (4.7)	103 (7.1)	0 (0.0)	0 (0.0)	7 (10.5)	5 (2.6)	1 (6.3)	5 (9.6)	1 (0.5)
LSD	1 (2.3)	26 (1.8)	0 (0.0)	0 (0.0)	1 (1.5)	0 (0.0)	0 (0.0)	2 (3.9)	0 (0.0)
Ecstasy in the form of a pill	1 (2.3)	53 (3.6)	0 (0.0)	0 (0.0)	0 (0.0)	0 (0.0)	0 (0.0)	3 (5.8)	0 (0.0)
Ecstasy in the form of a crystal (MDMA)	0 (0.0)	18 (1.2)	0 (0.0)	0 (0.0)	1 (1.5)	0 (0.0)	1 (6.3)	1 (1.9)	0 (0.0)
Ketamine	0 (0.0)	7 (0.5)	0 (0.0)	0 (0.0)	0 (0.0)	0 (0.0)	0 (0.0)	0 (0.0)	0 (0.0)
GHB/GBL	0 (0.0)	15 (1.0)	0 (0.0)	0 (0.0)	0 (0.0)	0 (0.0)	0 (0.0)	0 (0.0)	0 (0.0)
Amphetamines	1 (2.3)	7 (0.5)	0 (0.0)	0 (0.0)	0 (0.0)	0 (0.0)	0 (0.0)	0 (0.0)	0 (0.0)
Crack cocaine	2 (4.7)	27 (1.9)	0 (0.0)	0 (0.0)	0 (0.0)	1 (0.5)	0 (0.0)	1 (1.9)	0 (0.0)
Crystal Methamphetamines	0 (0.0)	62 (4.3)	0 (0.0)	0 (0.0)	0 (0.0)	0 (0.0)	0 (0.0)	0 (0.0)	0 (0.0)
Heroine	0 (0.0)	1 (0.1)	0 (0.0)	0 (0.0)	0 (0.0)	0 (0.0)	0 (0.0)	0 (0.0)	0 (0.0)
Mephedrone	0 (0.0)	0 (0.0)	0 (0.0)	0 (0.0)	0 (0.0)	0 (0.0)	0 (0.0)	0 (0.0)	0 (0.0)
Synthetic stimulants other than mephedrone (tusi)	0 (0.0)	6 (0.4)	0 (0.0)	0 (0.0)	0 (0.0)	0 (0.0)	0 (0.0)	1 (1.9)	1 (0.5)
Have taken drugs but not sure which	1 (2.3)	12 (0.8)	0 (0.0)	0 (0.0)	0 (0.0)	1 (0.5)	0 (0.0)	1 (1.9)	3 (1.5)
Have not consumed any drugs	16 (37.2)	551 (37.8)	16 (47.1)	32 (47.1)	34 (50.8)	113 (58.6)	4 (25.0)	22 (42.3)	123 (61.8)

The results of the descriptive analysis of the independent variables are presented below, based on the statistically significant differences observed between the study groups.

### Sociodemographic aspects

Overall, the group of people who practiced SDU and other participants comprised mainly young men with a mean age of 30 years (SD = 9.5), and a median age of 28 years (IQR = 12) (Tables 5–8). Most participants declared having completed university or postgraduate studies. The proportion of participants with postgraduate degrees was higher among those who practiced SDU (72.5%) than other participants (66.2%). A similar trend was observed at the country level in Bolivia, Brazil, Colombia, Ecuador, Guatemala, and Mexico. In both groups, a majority of participants were employed (71.3% and 67.2%, respectively). It was also observed that the proportion of individuals who felt comfortable or very comfortable with their economic status was high among those who did not practice SDU overall (40.4% vs. 42.1%) and country levels in Argentina, Brazil, Chile, Colombia, Mexico, and Paraguay. However, a remarkable difference between the groups was seen at country level (18.4% vs. 39.9%). Among migrants, a higher proportion of people who practiced SDU was observed, overall (5.6% vs. 4.1%) and in Argentina, Bolivia, Brazil, El Salvador, Panama, and Paraguay.

**Table 5:** Sociodemographic aspects of the men surveyed and association with sexualized drug use (SDU), in total sample and each country participating in LAMIS-2018 (part one).

	Sexualized drug use (last 12 months)														
	Yes n (%) No n (%) *P-value*														
VARIABLE	TOTAL SAMPLE			Argentina			Bolivia			Brazil			Chile		
	(N = 64655)			(N = 5504)			(N = 748)			(N = 18139)			(N = 4945)		
**Age** (mean)	30.2	29.7	*<0*.*01*	31.5	31.7	*0*.*580*	26.6	26.7	*0*.*901*	31.4	29.8	*<0*.*01*	29.3	30.7	*<0*.*01*
18–19	412 (4.7)	5419 (9.8)	*<0*.*01*	22 (3.1)	414 (8.7)	*<0*.*01*	5 (6.6)	116 (17.4)	*0*.*126*	88 /(3.7)	1303 (8.3)	*<0*.*01*	69 (5.8)	292 (7.8)	*<0*.*01*
20–29	4350 (50.0)	27282 (49.3)		317 (45.1)	1976 (41.6)		52 (69.3)	374 (56.2)		1041 (44.8)	7759 (49.5)		627 (52.6)	1741 (46.7)	
30–39	2765 (31.8)	14078 (25.4)		251 (35.7)	1323 (27.8)		15 (20.0)	121 (18.2)		811 (34.9)	4315 (27.5)		371 (31.1)	1077 (28.9)	
40–49	841 (9.6)	5605 (10.1)		83 (11.8)	687 (14.4)		2 (2.6)	29 (4.3)		256 (11.0)	1460 (9.3)		98 (8.2)	406 (10.9)	
50–59	282 (3.2)	2366 (4.2)		26 (3.7)	263 (5.5)		1 (1.3)	20 (3.0)		109 (4.7)	693 (4.4)		24 (2.0)	149 (4.0)	
60–69	34 (0.3)	448 (0.8)		4 (0.6)	76 (1.6)		0 (0.0)	5 (0.7)		14 (0.6)	113 (0.7)		1 (0.1)	52 (1.4)	
70 or more years	6 (0.1)	51 (0.1)		0 (0.0)	10 (0.2)		0 (0.0)	0 (0.0)		2 (0.1)	6 (0.0)		0 (0.0)	10 (0.2)	
**Migratory situation**															
Born in another country	489 (5.6)	2284 (4.1)	*<0*.*01*	119 (17.0)	478 (10.1)	*<0*.*01*	9 (12.1)	30 (4.5)	*<0*.*01*	46 (2.0)	151 (1.0)	*<0*.*01*	99 (8.3)	387 (10.4)	*0*.*037*
Born in country of residence	8193 (94.4)	52893 (95.9)		583 (83.0)	4267 (89.9)		65 (87.8)	633 (95.5)		2275 (98.0)	15481 (99.0)		1090 (91.7)	3335 (89.6)	
**Education level** (highest)															
No education or basic	56 (0.7)	615 (1.1)	*<0*.*01*	4 (0.6)	47 (1.0)	*0*.*211*	0 (0.0)	3 (0.5)	<0.01	20 (0.9)	250 (1.6)	<0.01	7 (0.6)	33 (0.9)	*0*.*482*
Secondary or high school	2328 (26.8)	18031 (32.7)		297 (42.2)	2126 (44.8)		12 (16.0)	231 (34.7)		525 (22.6)	4723 (30.2)		327 (27.5)	1058 (28.4)	
University or postgraduate	6294 (72.5)	36499 (66.2)		402 (57.2)	2568 (54.2)		63 (84.0)	431 (64.8)		1774 (76.5)	10652 (68.2)		856 (71.9)	2632 (70.7)	
**Current occupation**															
Employed	6182 (71.3)	37045 (67.2)	*<0*.*01*	521 (74.3)	3316 (69.9)	*<0*.*01*	41 (54.6)	367 (55.2)	*0*.*957*	1615 (69.6)	10173 (65.1)	*<0*.*01*	769 (64.6)	2455 (65.9)	*0*.*235*
Unemployed	830 (9.6)	5052 (9.1)		66 (9.4)	370 (7.8)		6 (8.0)	54 (8.1)		255 (11.0)	1813 (11.6)		122 (10.2)	311 (8.3)	
Student	1460 (16.8)	11652 (21.1)		97 (13.8)	930 (19.6)		27 (36.0)	229 (34.4)		379 (16.3)	3187 (20.4)		272 (22.8)	863 (23.1)	
Retired and others	204 (2.3)	1410 (2.6)		17 (2.4)	124 (2.6)		1 (1.3)	15 (2.2)		69 (2.9)	452 (2.9)		27 (2.2)	95 (2.5)	
**Feelings about your income**															
Really comfortable or comfortable	3518 (40.4)	23230 (42.1)	*<0*.*01*	251 (35.7)	2026 (42.7)	*<0*.*01*	29 (38.6)	250 (37.6)	*0*.*983*	925 (39.8)	6632 (42.4)	*<0*.*01*	516 (43.3)	1744 (46.8)	*<0*.*01*
Neither comfortable nor struggling	3410 (39.2)	22126 (40.0)		298 (42.3)	1948 (41.0)		34 (45.3)	308 (46.3)		864 (37.2)	5980 (38.2)		452 (37.9)	1445 (38.8)	
Really struggling or struggling	1762 (20.2)	9893 (17.9)		154 (21.9)	775 (16.3)		12 (16.0)	107 (16.1)		532 (22.9)	3037 (19.4)		222 (18.6)	538 (14.4)	

**Table 6:** Sociodemographic aspects of the men surveyed and association with sexualized drug use (SDU), in total sample and each country participating in LAMIS-2018 (part two).

	Sexualized drug use (last 12 months)														
	Yes n (%) No n (%) *P-value*														
VARIABLE	Colombia			Costa Rica			Ecuador			El Salvador			Guatemala		
	(N = 8208)			(N = 1012)			(N = 1440)			(N = 572)			(N = 1157)		
**Age** (mean)	28.3	28.6	*0*.*358*	30.2	30.4	*0*.*866*	28.0	27.9	*0*.*893*	29.6	28.2	*0*.*350*	30.0	28.1	*0*.*027*
18–19	86 (6.6)	832 (12.2)	*<0*.*01*	8 (4.5)	79 (9.6)	*0*.*140*	10 (7.0)	147 (11.4)	*0*.*312*	0 (0.0)	49 (9.1)	*0*.*421*	4 (3.8)	112 (10.8)	*0*.*186*
20–29	757 (58.0)	3566 (52.4)		92 (51.9)	391 (47.5)		85 (59.8)	712 (55.5)		17 (65.3)	297 (55.1)		58 (56.3)	581 (56.1)	
30–39	337 (25.8)	1448 (21.3)		53 (29.9)	203 (24.6)		37 (26.0)	288 (22.4)		8 /(30.7)	148 (27.4)		29 (28.1)	245 (23.6)	
40–49	106 (8.1)	686 (10.1)		17 (9.6)	91 (11.0)		6 (4.2)	98 (7.6)		0 (0.0)	33 (6.1)		7 (6.8)	58 (5.6)	
50–59	17 (1.3)	228 (3.3)		5 (2.8)	49 (5.9)		4 (2.8)	33 (2.5)		1 (3.8)	11 (2.0)		5 (4.8)	30 (2.9)	
60–69	0 (0.0)	34 (0.5)		2 (1.1)	9 (1.1)		0 (0.0)	4 (0.3)		0 (0.0)	1 (0.2)		0 (0.0)	9 (0.8)	
70 or more years	1 (0.1)	3 (0.0)		0 (0.0)	1 (0.1)		0 (0.0)	0 (0.0)		0 (0.0)	0 (0.0)		0 (0.0)	0 (0.0)	
**Migratory situation**															
Born in another country	64 (4.9)	336 (4.9)	*0*.*951*	10 (5.7)	70 (8.5)	*0*.*201*	20 (14.1)	148 (11.6)	*0*.*379*	3 (11.5)	11 (2.1)	*<0*.*01*	3 (2.9)	32 (3.1)	*0*.*919*
Born in country of residence	1240 (95.1)	6454 (95.1)		167 (94.3)	751 (91.5)		122 (85.9)	1131 (88.4)		23 (88.5)	526 (97.9)		100 (97.1)	1002 (96.9)	
**Education level** (highest)															
No education or basic	11 (0.8)	72 (1.1)	*<0*.*01*	1 (0.5)	32 (3.9)	*0*.*075*	2 (1.4)	12 (1.0)	*<0*.*01*	0 (0.0)	10 (1.9)	*0*.*714*	0 (0.0)	22 (2.1)	*<0*.*01*
Secondary or high school	410 (31.5)	2712 (39.9)		78 (44.1)	338 (41.1)		28 (19.7)	446 (34.9)		9 (34.6)	205 (38.0)		26 (25.2)	395 (38.3)	
University or postgraduate	881 (67.7)	4007 (59.0)		98 (55.4)	452 (55.0)		112 (78.9)	819 (64.1)		17 (65.4)	324 (60.1)		77 (74.8)	615 (59.6)	
**Current occupation**															
Employed	896 (68.9)	4404 (64.9)	*0*.*016*	137 (77.4)	596 (72.4)	*0*.*011*	83 (58.4)	731 (57.2)	*0*.*938*	22 (84.6)	337 (62.5)	*0*.*082*	84 (81.5)	709 (68.8)	*0*.*056*
Unemployed	144 (11.1)	754 (11.1)		15 (8.4)	45 (5.4)		17 (11.9)	144 (11.3)		3 (11.5)	64 (11.8)		6 (5.8)	124 (12.0)	
Student	233 (17.9)	1477 (21.7)		19 (10.7)	166 (20.1)		40 (28.1)	376 (29.4)		1 (3.8)	125 (23.2)		12 (11.6)	178 (17.2)	
Retired and others	27 (2.1)	149 (2.2)		6 (3.4)	16 (1.9)		2 (1.4)	26 (2.0)		0 (0.0)	13 (2.4)		1 (1.0)	20 (1.9)	
**Feelings about your income**															
Really comfortable or comfortable	532 (40.8)	2965 (43.6)	*<0*.*01*	82 (46.3)	394 (47.9)	*0*.*806*	45 (31.6)	458 (35.7)	*0*.*580*	4 (15.3)	167 (31.0)	*<0*.*01*	49 (47.5)	387 (37.4)	*0*.*074*
Neither comfortable nor struggling	489 (37.5)	2614 (38.5)		71 (40.1)	309 (37.5)		65 (45.7)	569 (44.4)		8 (30.7)	259 (48.0)		41 (39.8)	444 (42.9)	
Really struggling or struggling	283 (21.7)	1218 (17.9)		24 (13.5)	120 (14.6)		32 (22.5)	255 (19.9)		14 (53.8)	113 (21.0)		13 (12.6)	204 (19.7)	

**Table 7:** Sociodemographic aspects of the men surveyed and association with sexualized drug use (SDU), in total sample and each country participating in LAMIS-2018 (part three).

	Sexualized drug use (last 12 months)														
	Yes n (%) No n (%) *P-value*														
VARIABLE	Honduras			México			Nicaragua			Panamá			Paraguay		
	(N = 646)			(N = 14957)			(N = 534)			(N = 759)			(N = 591)		
**Age** (mean)	27.8	26.6	*0*.*305*	30.7	29.6	*<0*.*01*	23.7	26.2	*0*.*092*	31.1	29.4	0.193	28.4	26.9	*0*.*164*
18–19	2 (3.8)	71 (12.1)	*0*.*157*	88 (4.3)	1333 (10.4)	*<0*.*01*	1 (3.2)	84 (16.8)	*0*.*015*	3 (5.2)	76 (10.9)	*0*.*056*	5 (7.6)	76 (14.7)	*0*.*034*
20–29	30 (57.6)	361 (61.7)		963 (47.9)	6292 (49.2)		29 (93.5)	299 (59.9)		29 (50.8)	339 (48.9)		33 (50.7)	299 (57.9)	
30–39	15 (28.8)	117 (20.0)		674 (33.5)	3209 (25.1)		1 (3.2)	77 (15.4)		12 (21.0)	175 (25.2)		23 (35.3)	93 (18.0)	
40–49	4 (7.6)	18 (3.1)		210 (10.4)	1309 (10.2)		0 (0.0)	27 (5.4)		11 (19.3)	70 (10.1)		3 (4.6)	33 (6.4)	
50–59	1 (1.9)	17 (2.9)		65 (3.2)	528 (4.1)		0 (0.0)	10 (2.0)		1 (1.7)	31 (4.4)		1 (1.5)	13 (2.5)	
60–69	0 (0.0)	1 (0.1)		7 (0.3)	84 (0.6)		0 (0.0)	2 (0.4)		1 (1.7)	1 (0.1)		0 (0.0)	2 (0.4)	
70 or more years	0 (0.0)	0 (0.0)		2 (0.1)	14 (0.1)		0 (0.0)	0 (0.0)		0 (0.0)	1 (0.1)		0 (0.0)	0 (0.0)	
**Migratory situation**															
Born in another country	2 (3.9)	16 (2.7)	*0*.*645*	56 (2.8)	251 (2.0)	*0*.*016*	1 (3.2)	21 (4.2)	*0*.*789*	22 (38.6)	149 (21.6)	*<0*.*01*	7 (10.9)	18 (3.5)	*<0*.*01*
Born in country of residence	50 (96.1)	568 (97.3)		1949 (97.2)	12503 (98.0)		30 (96.8)	477 (95.8)		35 (61.4)	542 (78.4)		57 (89.1)	497 (96.5)	
**Education level** (highest)															
No education or basic	1 (1.9)	9 (1.5)	*0*.*961*	7 (0.3)	60 (0.5)	*<0*.*01*	0 (0.0)	4 (0.8)	*0*.*860*	1 (1.8)	4 (0.6)	*0*.*517*	0 (0.0)	7 (1.3)	*0*.*594*
Secondary or high school	15 (28.9)	176 (30.2)		405 (20.2)	3347 (26.2)		6 (19.3)	104 (20.9)		15 (26.3)	165 (23.8)		21 (32.8)	180 (34.9)	
University or postgraduate	36 (69.2)	398 (68.3)		1595 (79.5)	9346 (73.3)		25 (80.7)	390 (78.3)		41 (71.9)	523 (75.6)		43 (67.2)	329 (63.8)	
**Current occupation**															
Employed	32 (61.5)	348 (59.7)	*0*.*448*	1545 (77.1)	8926 (70.0)	*<0*.*01*	18 (58.1)	310 (62.2)	*0*.*572*	47 (82.4)	501 (72.4)	*0*.*406*	51 (79.7)	363 (70.5)	*0*.*197*
Unemployed	9 (17.3)	69 (11.8)		140 (6.9)	809 (6.3)		3 (9.7)	53 (10.6)		2 (3.5)	50 (7.2)		2 (3.1)	48 (9.3)	
Student	11 (21.1)	156 (26.7)		279 (13.9)	2694 (21.1)		9 (29.0)	131 (26.3)		7 (12.3)	128 (18.5)		9 (14.1)	96 (18.6)	
Retired and others	0 (0.0)	10 (1.7)		41 (2.0)	321 (2.5)		1 (3.2)	4 (0.8)		1 (1.7)	13 (1.9)		2 (3.1)	8 (1.6)	
**Feelings about your income**															
Really comfortable or comfortable	16 (30.7)	210 (35.9)	*0*.*624*	872 (43.4)	5816 (45.6)	*<0*.*01*	12 (38.7)	166 (33.3)	*0*.*822*	27 (47.3)	340 (49.0)	*0*.*945*	12 (18.4)	206 (39.9)	*<0*.*01*
Neither comfortable nor struggling	23 (44.2)	220 (37.6)		796 (39.6)	5228 (40.9)		13 (41.9)	225 (45.1)		22 (38.6)	252 (36.4)		35 (53.8)	212 (41.1)	
Really struggling or struggling	13 (25.0)	155 (26.5)		341 (16.9)	1725 (13.5)		6 (19.3)	108 (21.6)		8 (14.0)	101 (14.6)		18 (27.6)	98 (19.0)	

**Table 8:** Sociodemographic aspects of the men surveyed and association with sexualized drug use (SDU), in total sample and each country participating in LAMIS-2018 (part four).

	Sexualized drug use (last 12 months)											
	Yes n (%) No n (%) *P-value*											
**VARIABLE**	**Perú**			**Suriname**			**Uruguay**			**Venezuela**		
	(N = 2025)			(N = 216)			(N = 771)			(N = 2431)		
**Age** (mean)	30.5	29.3	*0*.*158*	31.5	30.9	*0*.*797*	31.9	32.2	*0*.*747*	29.1	31.6	*<0*.*01*
18–19	12 (7.5)	222 (12.1)	*0*.*079*	1 (4.3)	13 (6.9)	*0*.*984*	6 (4.4)	71 (11.3)	*0*.*019*	2 (1.6)	129 (5.7)	*<0*.*01*
20–29	73 (46.2)	895 (48.7)		11 (47.8)	86 (45.7)		68 (50.3)	250 (39.8)		68 (57.1)	1064 (46.9)	
30–39	47 (29.7)	429 (23.3)		6 (26.0)	57 (30.3)		35 (25.9)	150 (23.9)		40 (33.6)	603 (26.5)	
40–49	20 (12.6)	189 (10.3)		3 (13.0)	19 (10.1)		10 (7.4)	92 (14.6)		5 (4.2)	300 (13.2)	
50–59	3 (1.9)	87 (4.7)		2 (8.7)	11 (5.8)		14 (10.3)	49 (7.8)		3 (2.5)	144 (6.3)	
60–69	3 (1.9)	13 (0.7)		0 (0.0)	1 (0.5)		1 (0.7)	13 (2.0)		1 (0.8)	28 (1.2)	
70 or more years	0 (0.0)	2 (0.1)		0 (0.0)	1 (0.5)		1 (0.7)	2 (0.3)		0 (0.0)	1 (0.0)	
**Migratory situation**			* *			* *			* *			* *
Born in another country	14 (8.9)	106 (5.8)	*0*.*117*	2 (8.7)	8 (4.3)	*0*.*344*	9 (6.7)	35 (5.6)	*0*.*624*	3 (2.5)	37 (1.6)	*0*.*464 *
Born in country of residence	144 (91.1)	1730 (94.2)		21 (91.3)	180 (95.7)		126 (93.3)	592 (94.4)		116 (97.5)	2224 (98.4)	
**Education level** (highest)			* *			* *			* *			* *
No education or basic	0 (0.0)	12 (0.7)	*0*.*050 *	0 (0.0)	14 (8.5)	*0*.*331 *	0 (0.0)	11 (1.8)	*0*.*290 *	2 (1.7)	13 (0.6)	*0*.*330 *
Secondary or high school	53 (33.5)	776 (42.3)		8 (44.4)	80 (48.8)		56 (41.5)	262 (41.9)		37 (31.1)	707 (31.2)	
University or postgraduate	105 (66.5)	1045 (57.0)		10 (55.6)	70 (42.7)		79 (58.5)	352 (56.3)		80 (67.2)	1546 (68.2)	
**Current occupation**			* *			* *			* *			* *
Employed	114 (72.1)	1249 (68.2)	* 0*.*612*	19 (82.6)	137 (73.3)	*0*.*489*	102 (75.5)	437 (69.8)	*0*.*048*	86 (72.2)	1686 (74.3)	* 0*.*028 *
Unemployed	10 (6.3)	158 (8.6)		0 (0.0)	5 (2.6)		15 (11.1)	44 (7.0)		15 (12.6)	137 (6.0)	
Student	32 (20.2)	386 (21.1)		2 (8.7)	35 (18.7)		15 (11.1)	121 (19.3)		16 (13.4)	374 (16.5)	
Retired and others	2 (1.3)	39 (2.1)		2 (8.7)	10 (5.3)		3 (2.2)	24 (3.8)		2 (1.7)	71 (3.1)	
**Feelings about your income**			* *			* *			* *			* *
Really comfortable or comfortable	58 (36.7)	640 (34.8)	*0*.*815 *	14 (60.8)	112 (59.6)	*0*.*488*	45 (33.3)	247 (39.4)	*0*.*182*	29 (24.3)	470 (20.7)	*0*.*383 *
Neither comfortable nor struggling	71 (44.9)	874 (47.6)		8 (34.7)	53 (28.2)		69 (51.1)	266 (42.4)		51 (42.8)	920 (40.6)	
Really struggling or struggling	29 (18.3)	323 (17.6)		1 (4.3)	23 (12.2)		21 (15.5)	114 (18.2)		39 (32.7)	879 (38.7)	

### Socioepidemiological aspects

In terms of SDU practice, the percentage of people with some kind of sexual encounter with a casual partner during the last 12 months was higher in SDU practicing group than the other group (87.8% vs. 77.9%). This trend was observed in most countries (Tables 9–12). The number of casual sexual partners in the last year was also higher in people who practiced SDU; for example, 16.3% respondents who practiced SDU had 11–20 casual partners, whereas 7.9% people in the other group had 11–20 casual partners. A similar observation was made at the country level. Reports of having 11–20 casual sexual encounters without using a condom in the last year was also higher among people who practiced USD, at overall (4.9% vs. 1.6%) and country level, except Panama. A high level of satisfaction with their sexual life was more frequent among people who practiced SDU (52.7%) than the other participants (45%). A similar observation was made at the country level in Argentina, Brazil, Chile, Colombia, Mexico, and Peru. The number of people who received payments in exchange for having sex with men was higher among those who practiced SDU (13.4% vs. 6.7%). This difference was also observed in Argentina, Brazil, Chile, Colombia, Mexico, Panama, and Venezuela.

**Table 9:** Socioepidemiological aspects of the men surveyed and association with sexualized drug use (SDU), in total sample and each country participating in LAMIS-2018 (part one).

	Sexualized drug use (last 12 months)														
	Yes n (%) No n (%) *P-value*														
VARIABLE	TOTAL SAMPLE			Argentina			Bolivia			Brazil			Chile		
	(N = 64655)			(N = 5504)			(N = 748)			(N = 18139)			(N = 4945)		
**Have a steady partner** (currently)	2407 (27.8)	14129 (25.6)	*<0*.*01*	168 (23.9)	1051 (22.2)	*0*.*120*	21 (28.0)	164 (24.7)	*0*.*193*	619 (26.7)	3720 (23.8)	*<0*.*01*	407 (34.3)	1281 (34.5)	*0*.*172*
**Have any kind of sex with a non-steady male partner** (last 12 months)	7470 (87.2)	41054 (75.3)	*<0*.*01*	609 (87.6)	3674 (78.8)	*<0*.*01*	64 (85.3)	468 (71.1)	*<0*.*01*	2047 (89.7)	12122 (78.5)	*<0*.*01*	981 (83.1)	2555 (69.1)	*<0*.*01*
**Number of non-steady male sexual partners** (last 12 months)															
None	1326 (15.6)	16415 (30.3)	*<0*.*01*	105 (15.1)	1263 (27.2)	*<0*.*01*	14 (18.7)	214 (32.7)	*<0*.*01*	282 (12.5)	4077 (26.6)	*<0*.*01*	245 (20.9)	1389 (37.8)	*<0*.*01*
1–10	4287 (50.4)	30252 (55.9)		340 (49.0)	2634 (56.7)		37 (49.3)	378 (57.7)		1039 (46.0)	8291 (54.2)		621 (52.9)	1918 (52.2)	
11–20	1384 (16.3)	4293 (7.9)		115 (16.6)	413 (8.9)		11 (14.7)	37 (5.6)		434 (19.2)	1633 (10.7)		163 (13.9)	224 (6.1)	
More than 20	1506 (17.7)	3176 (5.9)		134 (19.3)	335 (7.2)		13 (17.3)	26 (4.0)		505 (22.3)	1304 (8.5)		145 (12.3)	144 (3.9)	
**Number of non-steady male partners with whom have had sex without a condom** (last 12 months)															
None	3195 (38.7)	30477 (57.6)	*<0*.*01*	278 (41.2)	2661 (58.4)	*<0*.*01*	30 (42.3)	381 (59.5)	*<0*.*01*	812 (36.7)	8269 (55.2)	*<0*.*01*	526 (46.4)	2270 (63.3)	*<0*.*01*
1–10	4231 (51.3)	21066 (39.8)		337 (50.0)	1768 (38.8)		37 (52.1)	250 (39.1)		1169 (52.8)	6162 (41.2)		517 (45.6)	1217 (34.0)	
11–20	409 (4.9)	845 (1.6)		33 (4.9)	80 (1.8)		2 (2.8)	5 (0.8)		110 (5.0)	329 (2.2)		51 (4.5)	58 (1.6)	
More than 20	420 (5.1)	552 (1.0)		26 (3.9)	45 (1.0)		2 (2.8)	4 (0.6)		121 (5.5)	213 (1.4)		40 (3.5)	38 (1.1)	
**Satisfaction with your sex life**															
Low (1–3)	569 (6.6)	5313 (9.6)	*<0*.*01*	32 (4.5)	376 (7.9)	*<0*.*01*	5 (6.7)	74 (11.1)	*0*.*470*	167 (7.2)	1825 (11.7)	*<0*.*01*	69 (5.8)	393 (10.5)	*<0*.*01*
Medium (4–7)	3540 (40.7)	25059 (45.4)		309 (44.0)	2327 (49.0)		39 (52.0)	341 (51.3)		1078 (46.4)	7761 (49.6)		540 (45.4)	1836 (49.3)	
High (8–10)	4581 (52.7)	24877 (45.0)		362 (51.5)	2046 (43.1)		31 (41.3)	250 (37.6)		1076 (46.4)	6063 (38.7)		581 (48.8)	1498 (40.2)	
**Getting paid to have sex with a man** (last 12 months)	1158 (13.4)	3606 (6.7)	*<0*.*01*	94 (13.5)	313 (6.7)	*<0*.*01*	14 (19.2)	72 (11.2)	*0*.*049*	224 (9.7)	731 (4.8)	*<0*.*01*	111 (9.4)	139 (3.8)	*<0*.*01*
**Self-reported HIV diagnosis**	2332 (27.0)	7843 (14.3)	*<0*.*01*	205 (29.4)	774 (16.4)	*<0*.*01*	9 (12.2)	78 (11.8)	*0*.*924*	611 (26.4)	2313 (14.8)	*<0*.*01*	334 (28.1)	601 (16.2)	*<0*.*01*
**Have ever taken PrEP**	288 (3.3)	715 (1.3)	*<0*.*01*	13 (1.9)	29 (0.6)	*<0*.*01*	1 (1.3)	3 (0.4)	*0*.*325*	116 (5.0)	278 (1.8)	*<0*.*01*	37 (3.1)	37 (1.0)	*<0*.*01*
**Positive diagnosis for other STIs** (at least once in lifetime)															
Hepatitis C	150 (1.7)	464 (0.8)	*<0*.*01*	7 (1.0)	42 (0.9)	*0*.*038*	1 (1.3)	2 (0.3)	*0*.*235*	41 (1.8)	146 (0.9)	*<0*.*01*	16 (1.3)	24 (0.6)	*0*.*040*
Syphilis	2352 (27.1)	7983 (14.5)	*<0*.*01*	203 (28.9)	817 (17.2)	*<0*.*01*	9 (12.0)	66 (9.9)	*0*.*812*	846 (36.5)	3273 (20.9)	*<0*.*01*	269 (22.6)	487 (13.1)	*<0*.*01*
Gonorrhea	1882 (21.7)	6452 (11.7)	*<0*.*01*	149 (21.2)	528 (11.1)	*<0*.*01*	13 (17.3)	88 (13.2)	*0*.*577*	530 (22.8)	1988 (12.7)	*<0*.*01*	237 (19.9)	447 (12.0)	*<0*.*01*
Chlamydia	658 (7.6)	2075 (3.8)	*<0*.*01*	57 (8.2)	177 (3.7)	*<0*.*01*	5 (6.7)	26 (3.9)	*0*.*458*	206 (8.9)	623 (4.0)	*<0*.*01*	68 (5.7)	163 (4.4)	*<0*.*01*
Condyloma	2173 (25.0)	8510 (15.4)	*<0*.*01*	202 (28.7)	898 (18.9)	*<0*.*01*	17 (22.7)	118 (17.7)	*0*.*576*	558 (24.0)	2307 (14.7)	*<0*.*01*	290 (24.4)	657 (17.6)	*<0*.*01*

**Table 10:** Socioepidemiological aspects of the men surveyed and association with sexualized drug use (SDU), in total sample and each country participating in LAMIS-2018 (part two).

	Sexualized drug use (last 12 months)														
	Yes n (%) No n (%) *P-value*														
VARIABLE	Colombia			Costa Rica			Ecuador			El Salvador			Guatemala		
	(N = 8208)			(N = 1012)			(N = 1440)			(N = 572)			(N = 1157)		
**Have a steady partner** (currently)	306 (23.5)	1615 (23.8)	*0*.*181*	50 (28.3)	202 (24.6)	*0*.*410*	42 (29.6)	318 (24.9)	*0*.*466*	7 (26.9)	148 (27.4)	*0*.*965*	35 (34.0)	288 (27.9)	*0*.*164*
**Have any kind of sex with a non-steady male partner** (last 12 months)	1125 (87.8)	5217 (77.9)	*<0*.*01*	150 (85.7)	572 (70.9)	*<0*.*01*	114 (81.4)	932 (73.4)	*0*.*040*	26 (100.0)	370 (69.2)	*<0*.*01*	91 (89.2)	749 (73.2)	*<0*.*01*
**Number of non-steady male sexual partners** (last 12 months)			* *			* *			* *			* *			* *
None	177 (13.9)	1828 (27.5)	*<0*.*01*	36 (20.7)	308 (38.3)	*<0*.*01*	31 (22.3)	395 (31.4)	*<0*.*01*	0 (0.0)	200 (37.7)	*<0*.*01*	16 (15.8)	338 (33.3)	*<0*.*01*
1–10	664 (52.3)	3963 (59.6)		102 (58.6)	433 (53.9)		72 (51.8)	732 (58.1)		21 (84.0)	282 (53.1)		49 (48.5)	593 (58.4)	
11–20	212 (16.7)	516 (7.7)		16 (9.2)	35 (4.3)		23 (16.5)	85 (6.7)		3 (12.0)	32 (6.0)		10 (9.9)	46 (4.5)	
More than 20	217 (17.1)	345 (5.2)		20 (11.5)	28 (3.5)		13 (9.4)	48 (3.8)		1 (4.0)	17 (3.2)		26 (25.7)	38 (3.7)	
**Number of non-steady male partners with whom have had sex without a condom** (last 12 months)			* *			* *			* *			* *			* *
None	439 (35.6)	3552 (54.5)	*<0*.*01 *	76 (45.0)	498 (63.5)	*<0*.*01*	55 (41.7)	731 (59.5)	*<0*.*01 *	3 (12.0)	288 (55.3)	*<0*.*01 *	34 (34.3)	553 (55.6)	*<0*.*01 *
1–10	666 (53.9)	2808 (43.1)		84 (49.7)	276 (35.2)		69 (52.3)	485 (39.5)		20 (80.0)	222 (42.6)		51 (51.5)	419 (42.2)	
11–20	63 (5.1)	90 (1.4)		6 (3.5)	5 (0.6)		7 (5.3)	10 (0.8)		1 (4.0)	9 (1.7)		6 (6.1)	15 (1.5)	
More than 20	67 (5.4)	65 (1.0)		3 (1.8)	5 (0.6)		1 (0.7)	2 (1.2)		1 (4.0)	2 (0.4)		8 (8.1)	7 (0.7)	
**Satisfaction with your sex life**			* *			* *			* *			* *			* *
Low (1–3)	95 (7.3)	597 (8.8)	*<0*.*01 *	17 (9.6)	95 (11.5)	*0*.*265 *	10 (7.0)	114 (8.9)	*0*.*524 *	0 (0.0)	57 (10.6)	*0*.*177 *	8 (7.8)	103 (9.9)	*0*.*172 *
Medium (4–7)	522 (40.0)	3085 (45.4)		66 (37.3)	346 (42.0)		58 (40.9)	559 (43.6)		13 (50.0)	212 (39.3)		38 (36.9)	459 (44.4)	
High (8–10)	687 (52.7)	3115 (45.8)		94 (53.1)	382 (46.4)		74 (52.1)	609 (47.5)		13 (50.0)	270 (50.1)		57 (55.3)	473 (45.7)	
**Getting paid to have sex with a man** (last 12 months)	238 (18.5)	611 (9.2)	*<0*.*01*	16 (9.2)	39 (4.8)	*0*.*022*	21 (14.9)	104 (8.3)	*0*.*010*	3 (11.5)	46 (8.7)	*0*.*625*	13 (12.6)	87 (8.6)	*0*.*178*
**Self-reported HIV diagnosis**	319 (24.6)	934 (13.8)	*<0*.*01*	32 (18.3)	87 (10.7)	*<0*.*01*	32 (22.7)	166 (13.0)	*<0*.*01*	2 (8.0)	64 (12.0)	*0*.*548*	17 (16.7)	107 (10.5)	*0*.*057*
**Have ever taken PrEP**	26 (2.0)	61 (0.9)	*<0*.*01*	4 (2.3)	4 (0.5)	*0*.*046*	2 (1.4)	16 (1.3)	*0*.*565*	1 (3.8)	3 (0.6)	*0*.*140*	5 (4.8)	11 (1.1)	*<0*.*01*
**Positive diagnosis for other STIs** (at least once in lifetime)			* *			* *			* *			* *			* *
Hepatitis C	22 (1.7)	50 (0.7)	*<0*.*01*	1 (0.6)	6 (0.7)	*0*.*960*	2 (1.4)	14 (1.1)	*0*.*570*	1 (3.8)	6 (1.1)	*0*.*471*	3 (2.9)	6 (0.6)	*0*.*023*
Syphilis	368 (28.3)	1014 (14.9)	*<0*.*01*	48 (27.3)	127 (15.5)	*<0*.*01*	27 (19.3)	118 (9.2)	*<0*.*01*	6 (23.1)	53 (9.8)	*0*.*088*	17 (16.7)	88 (8.5)	*0*.*020*
Gonorrhea	356 (27.4)	982 (14.5)	*<0*.*01*	43 (24.3)	112 (13.7)	*<0*.*01*	25 (17.9)	144 (11.3)	*0*.*026*	2 (7.7)	38 (7.1)	*0*.*838*	26 (25.5)	114 (11.1)	*<0*.*01*
Chlamydia	78 (6.0)	208 (3.1)	*<0*.*01*	21 (11.9)	36 (4.4)	*<0*.*01*	11 (7.9)	37 (2.9)	*<0*.*01*	0 (0.0)	13 (2.4)	*0*.*504*	6 (5.9)	38 (3.7)	*0*.*497*
Condyloma	307 (23.6)	1016 (15.0)	*<0*.*01*	38 (21.5)	107 (13.0)	*0*.*015*	26 (18.3)	156 (12.2)	*0*.*102*	8 (30.8)	79 (14.7)	*0*.*027*	25 (24.3)	153 (14.8)	*0*.*011*

**Table 11:** Socioepidemiological aspects of the men surveyed and association with sexualized drug use (SDU), in total sample and each country participating in LAMIS-2018 (part three).

	Sexualized drug use (last 12 months)														
	Yes n (%) No n (%) *P-value*														
VARIABLE	Honduras			México			Nicaragua			Panamá			Paraguay		
	(N = 646)			(N = 14957)			(N = 534)			(N = 759)			(N = 591)		
**Have a steady partner** (currently)	14 (26.9)	160 (27.4)	*0*.*994*	580 (29.0)	3372 (26.5)	*<0*.*01*	9 (29.0)	138 (27.8)	*0*.*978*	12 (21.4)	179 (25.9)	*0*.*312*	16 (25.0)	140 (27.2)	*0*.*929*
**Have any kind of sex with a non-steady male partner** (last 12 months)	42 (80.8)	376 (65.4)	*0*.*024*	1729 (87.6)	9463 (75.1)	*<0*.*01*	28 (90.3)	313 (63.5)	*<0*.*01*	45 (80.4)	488 (71.7)	*0*.*162*	59 (92.2)	365 (72.6)	*<0*.*01*
**Number of non-steady male sexual partners** (last 12 months)															
None	11 (21.2)	227 (39.7)	*<0*.*01*	300 (15.3)	3847 (30.7)	*<0*.*01*	5 (16.1)	209 (42.6)	*<0*.*01*	12 (21.4)	223 (33.1)	*<0*.*01*	6 (9.5)	171 (34.0)	*<0*.*01*
1–10	31 (59.6)	309 (54.0)		997 (50.9)	7198 (57.5)		18 (58.1)	265 (54.0)		32 (57.1)	389 (57.7)		33 (52.4)	280 (55.8)	
11–20	6 (11.5)	27 (4.7)		311 (15.9)	849 (6.8)		5 (16.1)	13 (2.6)		4 (7.1)	44 (6.5)		11 (17.5)	30 (6.0)	
More than 20	4 (7.7)	9 (1.6)		351 (17.9)	630 (5.0)		3 (9.7)	4 (0.8)		8 (14.3)	18 (2.7)		13 (20.6)	21 (4.2)	
**Number of non-steady male partners with whom have had sex without a condom** (last 12 months)															
None	22 (44.0)	336 (60.0)	*<0*.*01*	681 (36.0)	6993 (57.2)	*<0*.*01*	9 (32.1)	279 (58.1)	*<0*.*01*	25 (47.2)	404 (61.9)	*0*.*187*	20 (32.8)	280 (56.8)	*<0*.*0*
1–10	25 (50.0)	220 (39.3)		970 (51.3)	4925 (40.3)		16 (57.1)	199 (41.5)		27 (50.9)	240 (36.7)		31 (50.8)	200 (40.6)	
11–20	3 (6.0)	2 (0.3)		110 (5.8)	171 (1.4)		3 (10.7)	1 (0.2)		1 (1.9)	7 (1.1)		5 (8.2)	10 (2.0)	
More than 20	0 (0.0)	2 (0.3)		131 (6.9)	129 (1.1)		0 (0.0)	1 (0.2)		0 (0.0)	2 (0.3)		5 (8.2)	3 (0.6)	
**Satisfaction with your sex life**															
Low (1–3)	4 (7.7)	52 (8.9)	*0*.*790*	120 (6.0)	992 (7.8)	*<0*.*01*	2 (6.5)	63 (12.6)	*0*.*591*	4 (7.0)	66 (9.5)	*0*.*799*	2 (3.1)	47 (9.1)	*0*.*256*
Medium (4–7)	21 (40.4)	258 (44.1)		613 (30.5)	4734 (37.1)		13 (41.9)	201 (40.3)		27 (47.4)	330 (47.6)		30 (46.1)	226 (43.8)	
High (8–10)	27 (51.9)	275 (47.0)		1276 (63.5)	7043 (55.1)		16 (51.6)	235 (47.1)		26 (45.6)	297 (42.9)		33 (50.8)	243 (47.1)	
**Getting paid to have sex with a man** (last 12 months)	10 (19.2)	55 (9.8)	*0*.*034*	337 (16.9)	980 (7.9)	*<0*.*01*	6 (19.3)	50 (10.5)	*0*.*126*	12 (21.0)	53 (7.8)	*<0*.*01*	9 (14.1)	42 (8.5)	*0*.*145*
**Self-reported HIV diagnosis**	8 (15.4)	47 (8.1)	*0*.*074*	619 (31.0)	1661 (13.1)	*<0*.*01*	3 (9.7)	18 (3.6)	*0*.*096*	12 (21.0)	95 (13.8)	*0*.*133*	19 (29.2)	94 (18.3)	*0*.*037*
**Have ever taken PrEP**	2 (3.8)	8 (1.4)	*0*.*358*	65 (3.2)	200 (1.6)	*<0*.*01*	0 (0.0)	6 (1.2)	*0*.*537*	2 (3.5)	2 (0.3)	*<0*.*01*	2 (3.1)	4 (0.8)	*0*.*199*
**Positive diagnosis for other STIs** (at least once in lifetime)															
Hepatitis C	0 (0.0)	3 (0.5)	*0*.*530*	45 (2.2)	103 (0.8)	*<0*.*01*	0 (0.0)	8 (1.6)	0.022	2 (3.5)	12 (1.7)	*0*.*517*	1 (1.5)	9 (1.7)	*0*.*247*
Syphilis	7 (13.5)	34 (5.8)	*0*.*084*	420 (20.9)	966 (7.6)	*<0*.*01*	3 (9.7)	21 (4.2)	0.312	16 (28.1)	113 (16.4)	*0*.*062*	19 (29.2)	111 (21.6)	*0*.*248*
Gonorrhea	6 (11.5)	35 (6.0)	*0*.*238*	384 (19.2)	1283 (10.1)	*<0*.*01*	1 (3.2)	37 (7.5)	0.502	10 (17.5)	94 (13.6)	*0*.*166*	10 (15.4)	42 (8.2)	*0*.*151*
Chlamydia	1 (1.9)	10 (1.7)	*0*.*663*	158 (7.9)	521 (4.1)	*<0*.*01*	1 (3.2)	7 (1.4)	0.458	4 (7.0)	18 (2.6)	*<0*.*01*	3 (4.6)	17 (3.3)	*0*.*860*
Condyloma	8 (15.4)	60 (10.3)	*0*.*271*	572 (28.5)	1997 (15.7)	*<0*.*01*	6 (19.3)	58 (11.7)	0.387	7 (12.3)	88 (12.7)	*0*.*958*	21 (32.8)	87 (16.9)	*<0*.*01*

**Table 12:** Socioepidemiological aspects of the men surveyed and association with sexualized drug use (SDU), in total sample and each country participating in LAMIS-2018 (part four).

	Sexualized drug use (last 12 months)											
	Yes n (%) No n (%) *P-value*											
VARIABLE	Perú			Suriname			Uruguay			Venezuela		
	(N = 2025)			(N = 216)			(N = 771)			(N = 2431)		
**Have a steady partner** (currently)	43 (27.2)	415 (22.7)	*0*.*425*	8 (34.8)	74 (39.6)	*0*.*334*	42 (31.3)	213 (34.0)	*0*.*807*	28 (23.5)	651 (28.7)	*0*.*467*
**Have any kind of sex with a non-steady male partner** (last 12 months)	132 (84.1)	1333 (73.6)	*<0*.*01*	18 (78.3)	95 (52.2)	*0*.*018*	109 (80.7)	411 (66.4)	*<0*.*01*	101 (87.8)	1551 (69.3)	*<0*.*01*
**Number of non-steady male sexual partners** (last 12 months)												
None	29 (18.5)	550 (30.4)	*<0*.*01*	5 (21.7)	94 (52.2)	*<0*.*01*	32 (23.7)	246 (39.9)	*<0*.*01*	20 (17.4)	836 (37.5)	*<0*.*01*
1–10	76 (48.4)	980 (56.3)		15 (65.2)	81 (45.0)		69 (51.1)	291 (47.1)		71 (61.7)	1235 (55.4)	
11–20	27 (17.2)	157 (8.7)		0 (0.0)	4 (2.2)		14 (10.4)	48 (7.8)		19 (16.5)	100 (4.5)	
More than 20	25 (15.9)	119 (6.6)		3 (13.0)	1 (0.6)		20 (14.8)	32 (5.2)		5 (4.4)	57 (2.6)	
**Number of non-steady male partners with whom have had sex without a condom** (last 12 months)												
None	64 (41.3)	1016 (57.5)	*<0*.*01*	8 (36.4)	130 (74.7)	*<0*.*01*	64 (48.8)	392 (65.3)	*<0*.*01*	49 (43.7)	1444 (65.6)	*<0*.*01*
1–10	80 (51.6)	704 (39.8)		12 (54.5)	44 (25.3)		60 (45.8)	197 (32.8)		60 (53.6)	730 (33.1)	
11–20	3 (1.9)	26 (1.5)		0 (0.0)	0 (0.0)		4 (3.1)	9 (1.5)		1 (0.9)	18 (0.8)	
More than 20	8 (5.2)	21 (1.2)		2 (9.1)	0 (0.0)		3 (2.3)	2 (0.3)		2 (1.8)	11 (0.5)	
**Satisfaction with your sex life**												
Low (1–3)	16 (10.1)	175 (9.5)	*<0*.*01*	1 (4.4)	29 (15.4)	*0*.*329*	10 (7.4)	65 (10.4)	*0*.*044*	7 (5.9)	190 (8.4)	*0*.*408*
Medium (4–7)	62 (39.2)	970 (52.8)		13 (56.5)	87 (46.3)		46 (34.1)	269 (42.9)		52 (43.7)	1058 (46.6)	
High (8–10)	80 (50.6)	692 (37.7)		9 (39.1)	72 (38.3)		79 (58.5)	293 (46.7)		60 (50.4)	1021 (45.0)	
**Getting paid to have sex with a man** (last 12 months)	23 (14.8)	158 (8.9)	*0*.*016*	2 (8.7)	12 (6.9)	*0*.*746*	9 (6.7)	24 (4.0)	*0*.*169*	16 (13.4)	90 (4.1)	*<0*.*01*
**Self-reported HIV diagnosis**	57 (36.1)	344 (18.8)	*<0*.*01*	5 (22.7)	10 (5.3)	*<0*.*01*	25 (18.5)	68 (10.9)	*0*.*015*	23 (19.3)	382 (16.9)	*0*.*497*
**Have ever taken PrEP**	8 (5.1)	30 (1.6)	*<0*.*01*	2 (8.7)	1 (0.5)	*<0*.*01*	0 (0.0)	3 (0.5)	*0*.*649*	2 (1.7)	19 (0.8)	*0*.*570*
**Positive diagnosis for other STIs** (at least once in lifetime)												
Hepatitis C	4 (2.5)	15 (0.8)	*<0*.*01*	0 (0.0)	0 (0.0)	*-*	1 (0.7)	4 (0.6)	*0*.*921*	3 (2.5)	14 (0.6)	*0*.*035*
Syphilis	38 (24.0)	287 (15.6)	*0*.*022*	7 (30.4)	16 (8.5)	*<0*.*01*	27 (20.0)	72 (11.5)	*0*.*021*	22 (18.8)	320 (14.1)	*0*.*276*
Gonorrhea	47 (29.7)	262 (14.3)	*<0*.*01*	4 (17.4)	19 (10.1)	*0*.*386*	23 (17.0)	68 (10.9)	*0*.*131*	16 (13.6)	171 (7.6)	*0*.*036*
Chlamydia	17 (10.8)	101 (5.5)	*0*.*021*	5 (21.7)	6 (3.2)	*<0*.*01*	8 (5.9)	15 (2.4)	*<0*.*01*	9 (7.7)	59 (2.6)	*<0*.*01*
Condyloma	38 (24.0)	331 (18.0)	*0*.*133*	2 (8.7)	4 (2.1)	*0*.*181*	33 (24.4)	91 (14.5)	*0*.*018*	15 (12.7)	303 (13.4)	*0*.*503*

HIV diagnosis was self-reported by 27% people who practiced SDU, which was almost twofold higher than the other group (14.3%). This situation was also observed in Argentina, Brazil, Chile, Colombia, Costa Rica, Ecuador, Peru, Suriname, and Mexico; the greatest difference between the groups was observed in Mexico (31% vs. 13.1%). The prevalence of diagnosis of other STIs was reported by a higher proportion by people who practiced SDU than the other respondents, including hepatitis C (1.7% vs. 0.8%), chlamydia (7.6% vs. 3.8%), gonorrhea (21.7% vs. 11.7%), syphilis (27.1% vs. 14.5%), and condyloma (25% vs. 15.4%). At the country level, high prevalence rates were observed in people who practiced SDU in Brazil, Colombia, and Mexico, and the highest prevalence rates for syphilis and chlamydia were seen in Brazil (36.5%) and Paraguay (32.8%), respectively. The use of PrEP (sometime in life) was reported by a higher proportion of people who practiced SDU than the other respondents overall (3.3% vs. 1.3%) and in most countries.

### Psychosocial aspects

Severe anxiety-depression symptoms were more common among people practicing SDU, overall (9.2% vs. 7%) and in almost 50% participating countries (Tables 13–16). Greater social support was provided to people who practiced SDU, which was reflected in their higher scores in both subscales of social provision than the other group: reliable alliance (60.6% vs. 56.5%) and social integration (50.4% vs. 43.3%). Internalized homonegativity was registered with lower values in people who practiced SDU than the other respondents, both overall (1.3% vs. 1.7%) and in almost all countries. Episodes of homophobic intimidation were reported in a high proportion by people who practiced SDU overall (52.6% vs. 48.2%) and in Argentina, Brazil, Chile, and Mexico. Although homophobic insults were less prevalent in both groups, it was prevalent among people who practiced SDU overall (34.4% vs. 28.6%) and in Argentina, Brazil, Chile, Colombia, Mexico, and Costa Rica. Costa Rica had a more remarkable difference among the groups than the other countries (45.5% vs. 31.2%). Finally, a high proportion of homophobic aggression was reported among people who practiced SDU overall (4.1% vs. 3.0%), in Brazil (4.8% vs. 2.8%), and in Colombia (4.5% vs. 2.9%).

**Table 13:** Psychosocial aspects of the men surveyed and association with sexualized drug use (SDU), in total sample and each country participating in LAMIS-2018 (part one).

	Sexualized drug use (last 12 months)														
	Yes n (%) No n (%) *P-value*														
VARIABLE	TOTAL SAMPLE			Argentina			Bolivia			Brazil			Chile		
	(N = 64655)			(N = 5504)			(N = 748)			(N = 18139)			(N = 4945)		
**PHQ-4 scale** (anxiety & depression)			* *			* *						* *			* *
Normal	3069 (35.5)	24271 (44.3)	*<0*.*01*	222 (31.8)	1908 (40.5)	*<0*.*01*	18 (24.3)	206 (31.1)	*0*.*024 *	709 (30.6)	6194 (39.8)	*<0*.*01 *	442 (37.5)	1621 (43.8)	*<0*.*01 *
Mild	3657 (42.3)	20992 (38.3)		335 (48.0)	2029 (43.0)		32 (43.2)	335 (50.5)		913 (39.4)	5512 (35.4)		501 (42.5)	1450 (39.2)	
Moderate	1108 (12.8)	5694 (10.4)		92 (13.2)	518 (11.0)		20 (27.0)	92 (13.9)		351 (15.2)	1994 (12.8)		142 (12.0)	408 (11.0)	
Severe	795 (9.2)	3842 (7.0)		49 (7.0)	258 (5.5)		4 (5.4)	30 (4.5)		342 (14.8)	1852 (11.9)		95 (8.0)	224 (6.0)	
**Reliable partner subscale**			* *			* *			* *			* *			* *
Low (4–7)	224 (2.6)	1616 (2.9)	*<0*.*01*	13 (1.8)	118 (2.5)	*0*.*067*	2 (2.7)	43 (6.5)	*0*.*409*	36 (1.6)	350 (2.2)	*<0*.*01*	26 (2.2)	105 (2.8)	*0*.*162*
Medium (8–12)	3197 (36.8)	22406 (40.6)		232 (33.0)	1747 (36.8)		43 (57.3)	377 (56.7)		841 (36.2)	6109 (39.0)		391 (32.8)	1304 (35.0)	
High (13–16)	5269 (60.6)	31227 (56.5)		458 (65.2)	2884 (60.7)		30 (40.0)	245 (36.8)		1444 (62.2)	9190 (58.7)		773 (65.0)	2318 (62.2)	
**Social integration subscale**			* *			* *			* *			* *			* *
Low (4–7)	195 (2.2)	1726 (3.1)	*<0*.*01*	10 (1.4)	118 (2.5)	*<0*.*01*	2 (2.7)	45 (6.8)	*0*.*361*	30 (1.3)	395 (2.5)	*<0*.*01*	22 (1.8)	101 (2.7)	*<0*.*01*
Medium (8–12)	4114 (47.3)	29613 (53.6)		331 (47.1)	2456 (51.7)		55 (73.3)	455 (68.4)		1102 (47.5)	8139 (52.0)		493 (41.4)	1800 (48.3)	
High (13–16)	4381 (50.4)	23910 (43.3)		362 (51.5)	2175 (45.8)		18 (24.0)	165 (24.8)		1189 (51.2)	7115 (45.5)		675 (56.7)	1826 (49.0)	
**Internalized homonegativity** (0–6)			* *			* *			* *			* *			* *
n—mean	7695–1.3	45330–1.7	*<0*.*01*	597–1.3	3728–1.6	*<0*.*01*	54–2.3	507–2.5	*<0*.*01*	2129–1.1	13608–1.5	*<0*.*01*	1050–1.3	3108–1.6	*<0*.*01*
**Homophobic intimidation**			* *			* *			* *			* *			* *
Yes (last 12 months)	4564 (52.6)	26566 (48.2)	*<0*.*01*	347 (49.4)	2058 (43.4)	*<0*.*01 *	45 (60.0)	366 (55.0)	*0*.*412 *	1262 (54.4)	7590 (48.6)	*<0*.*01 *	585 (49.2)	1606 (43.1)	*<0*.*01 *
No (never or years ago)	4113 (47.4)	28593 (51.8)		355 (50.6)	2683 (56.6)		30 (40.0)	299 (45.0)		1056 (45.6)	8036 (51.4)		605 (50.8)	2118 (56.9)	
**Homophobic insults**			* *			* *			* *			* *			* *
Yes (last 12 months)	2986 (34.4)	15775 (28.6)	*<0*.*01*	208 (29.6)	1184 (25.0)	*<0*.*01*	24 (32.0)	232 (34.9)	*0*.*618*	920 (39.7)	5108 (32.7)	*<0*.*01 *	341 (28.7)	817 (21.9)	*<0*.*01 *
No (never or years ago)	5693 (65.6)	39363 (71.4)		495 (70.4)	3558 (75.0)		51 (68.0)	433 (65.1)		1397 (60.3)	10516 (67.3)		849 (71.3)	2908 (78.1)	
**Homophobic aggression**			* *			* *			* *			* *			* *
Yes (last 12 months)	357 (4.1)	1667 (3.0)	*<0*.*01*	15 (2.1)	131 (2.8)	*0*.*336*	0 (0.0)	30 (4.5)	*0*.*060*	110 (4.8)	433 (2.8)	*<0*.*01*	42 (3.5)	85 (2.3)	*0*.*018*
No (never or years ago)	8317 (95.9)	53477 (97.0)		688 (97.9)	4613 (97.2)		75 (100.0)	635 (95.5)		2205 (95.2)	15191 (97.2)		1148 (96.5)	3639 (97.7)	

**Table 14:** Psychosocial aspects of the men surveyed and association with sexualized drug use (SDU), in total sample and each country participating in LAMIS-2018 (part two).

	Sexualized drug use (last 12 months)														
	Yes n (%) No n (%) *P-value*														
VARIABLE	Colombia			Costa Rica			Ecuador			El Salvador			Guatemala		
	(N = 8208)			(N = 1012)			(N = 1440)			(N = 572)			(N = 1157)		
**PHQ-4 scale** (anxiety & depression)															
Normal	531 (41.1)	3400 (50.4)	*<0*.*01*	63 (35.6)	413 (50.9)	*<0*.*01*	53 (37.8)	611 (48.3)	*0*.*063*	7 (26.9)	215 (40.2)	*0*.*110*	40 (39.2)	412 (40.3)	*0*.*700*
Mild	553 (42.8)	2587 (38.4)		78 (44.1)	304 (37.4)		67 (47.8)	467 (36.9)		14 (53.9)	223 (41.7)		40 (39.2)	426 (41.7)	
Moderate	128 (9.9)	514 (7.6)		19 (10.7)	62 (7.6)		15 (10.7)	127 (10.0)		5 (19.2)	54 (10.1)		12 (11.8)	114 (11.2)	
Severe	81 (6.2)	243 (3.6)		17 (9.6)	33 (4.1)		5 (3.6)	60 (4.7)		0 (0.0)	43 (8.0)		10 (9.8)	69 (6.8)	
**Reliable partner subscale**															
Low (4–7)	48 (3.7)	210 (3.1)	*0*.*018*	7 (3.9)	26 (3.1)	*0*.*215*	4 (2.8)	63 (4.9)	*0*.*469*	3 (11.5)	24 (4.5)	*0*.*153*	5 (4.8)	58 (5.6)	*0*.*023*
Medium (8–12)	554 (42.5)	3170 (46.6)		79 (44.6)	315 (38.3)		74 (52.1)	626 (48.8)		10 (38.5)	281 (52.1)		32 (31.1)	459 (44.4)	
High (13–16)	702 (52.8)	3417 (50.3)		91 (51.4)	482 (58.6)		64 (45.1)	593 (46.3)		13 (50.0)	234 (43.4)		66 (64.1)	518 (50.0)	
**Social integration subscale**															
Low (4–7)	28 (2.1)	219 (3.2)	*<0*.*01*	11 (6.2)	27 (3.3)	*0*.*149*	7 (4.9)	60 (4.7)	*0*.*773*	2 (7.7)	19 (3.5)	*0*.*547*	4 (3.9)	55 (5.3)	*<0*.*01*
Medium (8–12)	697 (53.5)	4013 (59.0)		92 (52.0)	420 (51.0)		81 (57.0)	771 (60.1)		15 (57.7)	327 (60.7)		44 (42.7)	597 (57.7)	
High (13–16)	579 (44.4)	2565 (37.7)		74 (41.8)	376 (45.7)		54 (38.0)	451 (35.2)		9 (34.6)	193 (35.8)		55 (53.4)	383 (37.0)	
**Internalized homonegativity** (0–6)															
n—mean	1157–1.6	5655–1.9	*<0*.*01*	155–1.4	671–1.7	*0*.*021*	124–1.8	1000–2.1	*0*.*016*	24–2.1	407–2.1	*0*.*885*	89–1.6	849–2.1	*<0*.*01*
**Homophobic intimidation**															
Yes (last 12 months)	718 (55.1)	3539 (52.1)	*0*.*047*	96 (54.5)	408 (49.6)	*0*.*231*	76 (53.5)	639 (50.2)	*0*.*447*	17 (65.4)	255 (47.5)	*0*.*074*	53 (51.5)	540 (52.2)	*0*.*882*
No (never or years ago)	584 (44.9)	3247 (47.9)		80 (45.5)	415 (50.4)		66 (46.5)	635 (49.8)		9 (34.6)	282 (52.5)		50 (48.5)	494 (47.8)	
**Homophobic insults**															
Yes (last 12 months)	392 (30.1)	1558 (23.0)	*<0*.*01*	80 (45.5)	256 (31.2)	*<0*.*01*	44 (31.0)	315 (24.7)	*0*.*102*	14 (53.9)	201 (37.4)	*0*.*092*	37 (35.9)	348 (33.7)	*0*.*653*
No (never or years ago)	911 (69.9)	5220 (77.0)		96 (54.5)	565 (68.8)		98 (69.0)	961 (75.3)		12 (46.1)	336 (62.6)		66 (64.1)	684 (66.3)	
**Homophobic aggression**															
Yes (last 12 months)	59 (4.5)	196 (2.9)	*<0*.*01*	4 (2.3)	19 (2.3)	*0*.*975*	1 (0.7)	60 (4.7)	*0*.*026*	4 (15.4)	26 (4.8)	*0*.*019*	5 (4.9)	49 (4.8)	*0*.*963*
No (never or years ago)	1243 (95.5)	6587 (97.1)		172 (97.7)	803 (97.7)		141 (99.3)	1216 (95.3)		22 (84.6)	513 (95.2)		98 (95.1)	982 (95.2)	

**Table 15:** Psychosocial aspects of the men surveyed and association with sexualized drug use (SDU), in total sample and each country participating in LAMIS-2018 (part three).

	Sexualized drug use (last 12 months)														
	Yes n (%) No n (%) *P-value*														
VARIABLE	Honduras			México			Nicaragua			Panamá			Paraguay		
	(N = 646)			(N = 14957)			(N = 534)			(N = 759)			(N = 591)		
**PHQ-4 scale** (anxiety & depression)															
Normal	15 (28.8)	272 (47.1)	*<0*.*01*	768 (38.6)	6060 (47.9)	*<0*.*01*	10 (32.3)	232 (46.7)	*0*.*392*	26 (46.4)	361 (52.7)	*0*.*561*	16 (25.0)	150 (29.3)	*0*.*345*
Mild	31 (59.6)	209 (36.2)		837 (42.1)	4800 (37.9)		14 (45.2)	193 (38.8)		24 (42.9)	251 (36.6)		28 (43.7)	241 (47.1)	
Moderate	2 (3.8)	70 (12.1)		236 (11.9)	1143 (9.0)		5 (16.1)	48 (9.7)		5 (8.9)	45 (6.6)		14 (21.9)	69 (13.5)	
Severe	4 (7.7)	26 (4.5)		148 (7.4)	654 (5.2)		2 (6.4)	24 (4.8)		1 (1.8)	28 (4.1)		6 (9.4)	52 (10.1)	
**Reliable partner subscale**															
Low (4–7)	3 (5.8)	30 (5.1)	*0*.*823*	58 (2.9)	373 (2.9)	*0*.*106*	0 (0.0)	21 (4.2)	*0*.*092*	2 (3.5)	26 (3.8)	*0*.*189*	4 (6.1)	22 (4.2)	*0*.*099*
Medium (8–12)	26 (50.0)	270 (46.2)		703 (35.0)	4777 (37.4)		12 (38.7)	265 (53.1)		15 (26.3)	265 (38.2)		26 (40.0)	279 (54.1)	
High (13–16)	23 (44.2)	285 (48.7)		1248 (62.1)	7619 (59.7)		19 (61.3)	213 (42.7)		40 (70.2)	402 (58.0)		35 (53.9)	215 (41.7)	
**Social integration subscale**															
Low (4–7)	3 (5.8)	22 (3.8)	*0*.*345*	60 (3.0)	429 (3.4)	*<0*.*01*	0 (0.0)	21 (4.2)	*0*.*364*	0 (0.0)	32 (4.6)	*0*.*159*	1 (1.5)	31 (6.0)	*<0*.*01*
Medium (8–12)	25 (48.1)	340 (58.1)		899 (44.7)	6544 (51.2)		19 (61.3)	326 (65.3)		29 (50.9)	379 (54.7)		30 (46.2)	333 (64.5)	
High (13–16)	24 (46.1)	223 (38.1)		1050 (52.3)	5796 (45.4)		12 (38.7)	152 (30.5)		28 (49.1)	282 (40.7)		34 (52.3)	152 (29.5)	
**Internalized homonegativity** (0–6)															
n—mean	46–1.6	451–2.4	*<0*.*01*	1770–1.4	10197–1.7	*<0*.*01*	23–2.0	370–2.2	*0*.*451*	49–1.6	553–2.1	*<0*.*01*	56–1.6	384–2.1	*<0*.*01*
**Homophobic intimidation**															
Yes (last 12 months)	33 (63.5)	338 (58.1)	*0*.*450*	998 (49.8)	5865 (46.0)	*<0*.*01*	17 (54.8)	299 (60.0)	*0*.*567*	32 (56.1)	345 (49.9)	*0*.*367*	38 (59.4)	282 (54.6)	*0*.*474*
No (never or years ago)	19 (36.5)	244 (41.9)		1007 (50.2)	6890 (54.0)		14 (45.2)	199 (40.0)		25 (43.9)	346 (50.1)		26 (40.6)	234 (45.4)	
**Homophobic insults**															
Yes (last 12 months)	25 (48.1)	227 (39.0)	*0*.*200*	672 (33.5)	3623 (28.4)	*<0*.*01*	18 (60.0)	206 (41.4)	*0*.*046*	23 (40.4)	184 (26.7)	*0*.*027*	30 (46.9)	205 (39.7)	*0*.*272*
No (never or years ago)	27 (51.9)	355 (61.0)		1334 (66.5)	9130 (71.6)		12 (40.0)	291 (58.6)		34 (59.6)	506 (73.3)		34 (53.1)	311 (60.3)	
**Homophobic aggression**															
Yes (last 12 months)	3 (5.8)	21 (3.6)	*0*.*434*	82 (4.1)	424 (3.3)	*0*.*080*	1 (3.2)	18 (3.6)	*0*.*910*	3 (5.3)	14 (2.0)	*0*.*114*	5 (7.8)	16 (3.1)	*0*.*058*
No (never or years ago)	49 (94.2)	561 (96.4)		1922 (95.9)	12323 (96.7)		30 (96.8)	480 (96.4)		54 (94.7)	678 (98.0)		59 (92.2)	499 (96.9)	

**Table 16:** Psychosocial aspects of the men surveyed and association with sexualized drug use (SDU), in total sample and each country participating in LAMIS-2018 (part four).

	Sexualized drug use (last 12 months)											
	Yes n (%) No n (%) *P-value*											
VARIABLE	Perú			Suriname			Uruguay			Venezuela		
	(N = 2025)			(N = 216)			(N = 771)			(N = 2431)		
**PHQ-4 scale** (anxiety & depression)												
Normal	51 (32.7)	736 (40.6)	*0*.*111*	16 (69.6)	111 (59.4)	*0*.*491*	51 (37.8)	280 (44.8)	*0*.*346*	31 (26.3)	1089 (48.7)	*<0*.*01*
Mild	76 (48.7)	836 (46.1)		4 (17.4)	60 (32.1)		59 (43.7)	248 (39.7)		51 (43.2)	821 (36.7)	
Moderate	21 (13.5)	159 (8.7)		2 (8.7)	9 (4.8)		18 (13.3)	60 (9.6)		21 (17.8)	208 (9.3)	
Severe	8 (5.1)	83 (4.6)		1 (4.3)	7 (3.7)		7 (5.2)	37 (5.9)		15 (12.7)	119 (5.3)	
**Reliable partner subscale**												
Low (4–7)	8 (5.1)	67 (3.6)	*0*.*228*	0 (0.0)	3 (1.6)	*0*.*809*	4 (3.0)	15 (2.4)	*0*.*179*	1 (0.8)	62 (2.7)	*0*.*109*
Medium (8–12)	70 (44.3)	937 (51.0)		13 (56.5)	100 (53.2)		37 (27.4)	224 (35.7)		39 (32.8)	901 (39.7)	
High (13–16)	80 (50.6)	833 (45.4)		10 (43.5)	85 (45.2)		94 (69.6)	388 (61.9)		79 (66.4)	1306 (57.6)	
**Social integration subscale**												
Low (4–7)	6 (3.8)	63 (3.4)	*0*.*158*	1 (4.4)	3 (1.6)	*0*.*568*	4 (3.0)	18 (2.9)	*0*.*047*	4 (3.4)	68 (3.0)	*0*.*309*
Medium (8–12)	84 (53.2)	1119 (60.9)		15 (65.2)	115 (61.2)		50 (37.0)	305 (48.6)		53 (44.5)	1174 (51.7)	
High (13–16)	68 (43.0)	655 (35.7)		7 (30.4)	70 (37.2)		81 (60.0)	304 (48.5)		62 (52.1)	1027 (45.3)	
**Internalized homonegativity** (0–6)												
n—mean	139–1.7	1470–2.1	*<0*.*01*	19–1.8	143–1.9	*0*.*699*	116–1.1	492–1.4	*0*.*059*	98–1.4	1737–1.8	*<0*.*01*
**Homophobic intimidation**												
Yes (last 12 months)	99 (62.7)	1016 (55.5)	*0*.*084*	9 (39.1)	69 (36.9)	*0*.*834*	73 (54.1)	279 (44.6)	*0*.*045*	66 (55.9)	1072 (47.3)	*0*.*068*
No (never or years ago)	59 (37.3)	813 (44.5)		14 (60.9)	118 (63.1)		62 (45.9)	347 (55.4)		52 (44.1)	1193 (52.7)	
**Homophobic insults**												
Yes (last 12 months)	67 (42.4)	612 (33.5)	*0*.*024*	8 (34.8)	43 (23.0)	*0*.*213*	48 (35.6)	177 (28.3)	*0*.*093*	35 (29.4)	479 (21.2)	*0*.*034*
No (never or years ago)	91 (57.6)	1214 (66.5)		15 (65.2)	144 (77.0)		87 (64.4)	449 (71.7)		84 (70.6)	1782 (78.8)	
**Homophobic aggression**												
Yes (last 12 months)	10 (6.4)	76 (4.2)	*0*.*192*	2 (8.7)	4 (2.1)	*0*.*075*	4 (3.0)	12 (1.9)	*0*.*444*	7 (5.9)	53 (2.3)	*0*.*016*
No (never or years ago)	147 (93.6)	1751 (95.8)		21 (91.3)	183 (97.9)		131 (97.0)	613 (98.1)		112 (94.1)	2210 (97.7)	

## Discussion

The LAMIS-2018 was the first survey to describe the SDU phenomenon in a large population of gay men and other MSM from LA countries, addressing multiple aspects of the psycho-socio-sexual health in 18 countries of the region. Literature has revealed that gay men may be more prone to excessive alcohol consumption and drug use than the general male population [41], in part due to the discrimination and stigma experienced by sexual minorities, which have been associated with increased alcohol and drug use [42]. This study revealed high percentage of alcohol consumption and dependence (90.1% and 21.2%, respectively), which exceeded the levels reported by the WHO for the general population of the American region. Considering that the abuse of most illegal drugs, including methamphetamine, cannabis, cocaine, heroin, and polydrugs, generally occur in conjunction with alcohol, intervention strategies should not exclude legal drugs in their communication aspects, especially considering the risks associated with polydrug use and the interaction between PS [43]. Cannabis was predominantly used by the surveyed population. This observation was consistent with the previously observed trend in the region [2, 18]. The highest prevalence of cannabis use was observed in Chile (49.9%), which is the leading LA country in terms of cannabis consumption and drug abuse/dependence rates [44]. Consumption of synthetic drugs was registered at a low extent in the studied population; however, the use of synthetic cannabinoids have become increasingly common [45]. Ecstasy is the second most commonly used synthetic drug, which has experienced a global boom owing to cultural influence among the young population [46]. The use of injectable drugs was reported by a low proportion of the participants, which was in accordance with the evidence found for the general population of the region in previous reports [21] and research papers [47]. However, it is necessary to constantly monitor the use of injectable drugs in the sexual context, as the practice of slamsex has been increasing in other regions along with the corresponding risks for infection transmission by parenteral route while sharing the injection material [25].

The prevalence of SDU (last 12 months) in this study was relatively similar to that reported in the EMIS-2017 conducted in the European region (13.6% vs. 10.4%) ([35]; however, the prevalence in this study was lower than the reported prevalence in an investigation carried out in Mexico (23.9%) [48], though there are certain differences in the definition of the phenomenon that hinders the comparability of the studies. Chile had the highest prevalence of SDU (24.2%), exceeding the general prevalence by almost 10 points (13.6%). The prevalence of SDU with multiple partners and the proportion of preferring private homes for such encounters was similar to that reported by the EMIS-2017 study (6.6% vs. 6.7%); the increased use of private homes for group sexual encounters is attributable to the use of mobile dating applications [49]. In group encounters, a predominance of the cannabis-poppers-Viagra triad was observed, which are associated with the sexual context in MSM and heterosexual population as these drugs can facilitate penetrative practices and prolong the sexual intercourse [50, 51]. It was found that people who practiced SDU had higher educational qualifications than the other respondents of the study (72.5% vs. 66.2%), as opposed to the general prejudice of marginality historically associated with drug use [52]. High proportion of sexual behaviors that can increase exposure to HIV/STIs and of HIV diagnosis among people who practiced SDU observed in this study are in line with the literature. It has been reported that a higher prevalence of HIV/STIs and greater tendency to develop sexual behaviors that render them more vulnerable to STIs are seen in a segment of people who use drugs to enhance and prolong their sexual relations [53, 54]. It is important to mention the case of Mexico where a remarkable difference was observed in the self-reported HIV diagnosis among people who practiced SDU and those who did not (31% vs. 13.1%), suggesting a possible relationship between drug use and a higher prevalence of HIV; similar observation was made in the general population of the region [55]. Similarly, the self-reported HIV diagnosis in Chile was 28.1% among those who practiced SDU, indicating the re-emergence of HIV in this country, wich presents the highest increase in the number of cases in the last 10 years within the LA region [56, 57]. This phenomenon is directly related to the lack of preventive public policies [58].

This research revealed a low prevalence of PrEP use among those who practiced SDU (3.3%) and non-users (1.3%). This observation is in contrast with the phenomenon observed in other regions such as the UK, wherein usage rates exceed 20% [59], and France, where programs have been implemented at a national level to deliver PrEP services to the groups who are most likely at risk of HIV infection [60]. However, it is necessary to consider that PrEP has only recently been available in LA, and mainly linked to studies conducted in key populations, such as the “ImPrEP Project”, that addressed strategic aspects for the implementation of PrEP in integrated public health services in Brazil, Mexico, and Peru [61]. In Chile, a pilot study on the implementation of PrEP as a public health policy has been conducted in some regions of the country since 2019, but it does not identify people who practice SDU as a target group [62] despite the fact that PrEP has become a feasible strategy for HIV prevention in these population [63, 64].

People who practiced SDU reported high levels of satisfaction with their sexual life, which is consistent with the increased sexual enjoyment and less anxiety reported by people who use drugs in the sexual context [65]. On the other hand, payment in exchange for sex with men was high among people who practiced SDU than the other respondents (13.4% vs. 6.7%), indicating that the phenomenon of transactional sex is frequently observed in gay men, other MSM and trans women in relation with alcohol and drugs consumption and psychosocial factors, so these people constitute a particularly vulnerable group that needs to be prioritized while developing intervention strategies [66]. Another differential aspect observed in this study was the higher prevalence of severe anxiety/depression symptoms in people who practiced SDU, which in line with multiple studies that have revealed a greater susceptibility to depression, anxiety and/or drug dependence in MSM who practice SDU [67]. The impact of the phenomena of stigma and persistent homophobia in LA region can also affect the mental health of these population [68], as evidenced by the high percentages of intimidation (52.6%) and homophobic insults (34.4%) reported by people practicing SDU, especially considering that these experiences of homophobic bullying have been described as a risk factor for increased substance use in young adults, especially among victims with depressive symptoms [69]. On the other hand, the higher levels of perceived social support among those who reported drug use in the sexual context, as well as a lower internalized homonegativity, could be related to the fact that those who have an active social life, who attend parties and who are in permanent connection with their peers through the use of social networks or dating applications, could be more inclined to experiment with practices such as drug use during their sexual encounters [70]. Drug use can be a creative or experimental response of people and not necessarily a problem in all cases [71].

### Limitations

LAMIS-2018 was conducted using an online questionnaire, implying that the participants had a certain level of knowledge and access to mobile or desktop devices and the Internet. However, it was not a major obstacle among the population of gay men and other MSM [72]. Besides, the promotion was mainly done on web pages, social networks, and virtual communities frequently visited by MSM; therefore, the populations with limited resources, residents in areas with little access to the Internet, people not integrated in the LGTBIQ+ community, and people who did not frequently use virtual tools were underrepresented; therefore, LAMIS-2018 is likely to have covered a younger population with a higher level of education and employment rate than the general population of each participating country. Finally, in LAMIS-2018 PrEP use was measured for "lifetime" and not for a more recent period, such as "in the past 12 months".

## Conclusions

SDU practice was reported by a high percentage of the people surveyed in LAMIS-2018, wherein a predominance of drugs related to sexual practices (poppers, Viagra) and other related to recreational use like cannabis was observed. This SDU phenomenon is consistent with the availability of substances and the typical consumption profile in the LA context, and needs to be made visible as a public health problem in the region. The aspects described in this study, such as the higher proportion of self-reported HIV diagnosis and severe symptoms of anxiety-depression among those who practiced SDU, show that in order to reduce the harmful impacts that can sometimes result from the use of drugs in the sexual context, the implementation of combined preventive strategies adapted to each country is essential. This strategies must have the active participation of the most exposed communities, through community outreach programs and peer education, using technology to improve the reach of preventive efforts in the target population. Facilitating access to PrEP through dispensing from community organizations is key. Finally, given the multidimensional nature of the phenomenon, it is necessary to develop health policies that address drug use from a harm reduction perspective and promote access to mental health services and support in situations of homophobia and stigma, from a transdisciplinary, inclusive perspective and with an approach based on human rights.
